# A novel human specific lncRNA MEK6-AS1 regulates adipogenesis and fatty acid biosynthesis by stabilizing *MEK6* mRNA

**DOI:** 10.1186/s12929-024-01098-3

**Published:** 2025-01-08

**Authors:** Di Li, Yunhua Chen, Xingyu Zhu, Yanlei Yang, Hongling Li, Robert Chunhua Zhao

**Affiliations:** 1 Institute of Basic Medical Sciences Chinese Academy of Medical Sciences, School of Basic Medicine, Peking Union Medical College, Beijing, China; 2 Center for Excellence in Tissue Engineering, Chinese Academy of Medical Sciences, Beijing, China; 3 State Key Laboratory of Common Mechanism Research for Major Diseases, Chinese Academy of Medical Sciences, Beijing, China; 4 Beijing Key Laboratory of New Drug Development and Clinical Trial of Stem Cell Therapy (BZ0381), Beijing, China; 5 Clinical Biobank, Department Medical Research Central, Peking Union Medical College Hospital, Chinese Academy of Medical Sciences and Peking Union Medical College, Beijing, China

**Keywords:** Obesity, Adipogenesis, Adipogenic differentiation, Hepatic steatosis organoid, Metabolic associated fatty liver disease, Mesenchymal stem cell, LncRNA, MEK6-AS1, *MEK6*

## Abstract

**Background:**

Obesity is becoming one of the major non-communicable diseases with increasing incidence and risks that cannot be ignored. However effective and safe clinical treatment strategies still need to be deeply explored. Increased number and volume of adipocytes lead to overweight and obesity. The aim of our work is to identify lncRNAs that have important regulatory in differentiation of human mesenchymal stem cells (MSCs) into adipocytes, and to provide effective targets for clinical prevention and treatment of obesity and related metabolic disorders.

**Methods:**

We extracted primary MSCs from human adipose tissue, and conducted expression profile analysis of lncRNAs during adipogenic differentiation of MSCs to screen changed lncRNAs. Characteristics of lncRNA were revealed mainly by RACE and RNA FISH. Loss- and gain-of function experiments in vivo and in vitro were used to analyze effects of lncRNA. Targeted metabolomics was utilized to detect levels of free fatty acids. RNA pull-down, mRNA stability tests, etc. were employed to explore mechanisms of lncRNA.

**Results:**

Human-specific lncRNA, we named it MEK6-AS1, was the most up-regulated transcript during adipogenic differentiation of MSCs. MEK6-AS1 was highly expressed in adipose tissue samples from individuals with BMI ≥ 25 and positively correlated with adipogenic marker genes in these samples. Knocking down lncRNA inhibited expression of adipogenic differentiation markers and ectopic adipogenesis, reducing contents of various free fatty acids, as well as promoting osteogenic differentiation. Overexpression of lncRNA had the opposite effects to the above processes. We also found that MEK6-AS1 was elevated during hepatic steatosis organoid generation. Mechanistically, MEK6-AS1 worked partially through stabilization of *MEK6* mRNA by NAT10.

**Conclusions:**

We have identified a human-specific lncRNA (MEK6-AS1) with position information in the genomic database but has not been extensively reported. We demonstrated that MEK6-AS1 as a novel lncRNA involved in adipogenic differentiation and adipogenesis, fatty acid metabolism, and osteogenic differentiation. We found that MEK6-AS1 may exert its effect by enhancing *MEK6* mRNA stability through NAT10. Our study may provide insights into implication of lncRNAs in stem cell biology and offer a new potential therapeutic target for the prevention and treatment of obesity and other related disease.

**Supplementary Information:**

The online version contains supplementary material available at 10.1186/s12929-024-01098-3.

## Background

Overweight and obesity is defined by the World Health Organization as abnormal or excessive fat accumulation that can impair health [1]. More and more information warn that obesity has become a global pandemic of chronic disease, and the obese population is gradually getting younger [2, 3]. Intrinsic considerations such as genetic, epigenetic, and endocrine factors, interacting with environmental factors are now believed to contribute to theprevalence of obesity [4, 5].

Individuals with obesity may experience chronic inflammation over an extended period [6, 7]. Due to metabolic and endocrine disorders, they are at a higher risk of developing diseases such as type II diabetes, metabolic associated fatty liver disease (MAFLD), cardiovascular diseases and tumors [8–11]. We also need to consider the association between COVID-19 and obesity pandemic in the future [12–14]. However, there are still many challenges to safe treatment of obesity. Individuals with severe obesity are not suitable candidates for weight loss through intensive exercise [15]. Weight-loss drugs like rimonabant, sibutramine and lorcaserin have been withdrawn from international markets due to side effects [16]. Nowadays GLP-1 receptor agonists have also been reported rebound effects [17]. In summary, there is an urgent need to explore the mechanisms underlying obesity to generate new ideas for the clinical challenges.

Obesity is mainly caused by pathological expansion of adipocytes in the volume and/or number. Adipocytes are mainly developed from mesenchymal stem cells (MSCs) by graded differentiation [18]. The complex process is finely regulated by classical transcription factors and signaling pathways, such as CCAAT enhancer binding protein alpha (CEBPA), peroxisome proliferator activated receptor gamma (PPARG), Wnt/β-catein pathway, TGF-β/BMP pathway, etc. [19–21]. Abnormal adipogenesis process is closely related to the development of obesity [22–24]. However, the full scope of transcription factors and pathways involved in adipogenic differentiation of MSCs remains to be well explored, especially in the area of epigenetics such as non-coding RNAs [5, 25].

In the mammalian genome, 4–9% of the sequences-producing transcripts are long non-coding RNAs (lncRNAs) which are longer than 200nt and rarely encode proteins [26]. LncRNAs can regulate gene expression at various levels, including chromatin structure, transcriptional level, and post-transcriptional level, thereby being closely associated with numerous physiological and pathological procedures [27]. Many lncRNAs have been reported to be crucial for many biological processes that contribute to obesity, such as adipogenic differentiation, adipogenesis, lipid metabolism, etc. [28–31]. For instance, lncRNA-Adi which interacted with microRNA-449a to promote translation of cyclin-dependent kinase 6, and to activate pRb-E2F1 pathway to enhance adipogenesis [28]. Absence of lncRNA LIPTER in human cardiomyocytes impaired lipid transport, mitochondrial function, and survival of human induced pluripotent stem cell-derived cardiomyocytes through NK2 Homeobox 5 gene-LIPTER axis [29]. Piero Carninci et al. generated an atlas of 27,919 human lncRNA genes across 1829 primary human cell types and tissue samples, identifying 19,175 potentially functional lncRNAs within the human genome in 2017 [32]. LncRNAWiki 2.0 (https://ngdc.cncb.ac.cn/lncrnawiki), currently one of the largest knowledge bases of human lncRNAs, has so far cataloged only 2609 experimentally validated lncRNAs [33]. Among these lncRNAs, those associated with tumors account for 85.86%, with most lncRNAs in the database already demonstrating well-defined regulatory mechanisms. In contrast, lncRNAs related to obesity account for only 3.67%, and those associated with diabetes for 5.27%. It is still imperative to thoroughly investigate the effects and mechanisms of novel lncRNAs especially conserved in human on obesity and related diseases.

In this study, we used human adipose derived-MSCs (hAMSCs) rather than other sources to investigate the mechanisms of adipogenic differentiation because which can better mimic the process of adipogenesis in vivo. We performed expression profiling analysis of lncRNAs in hAMSCs before and after adipogenic differentiation, and identified lncRNA MEK6-AS1 as the most significantly up-regulated one, which is significantly elevated during both adipogenesis and hepatic steatosis organoid formation (an organoid models for the study of MAFLD generation). Expression analysis of a certain number of samples, as well as in vivo tests indicated the correlation between MEK6-AS1 and obesity to some extent. We also demonstrated the role of MEK6-AS1 as a novel lncRNA in regulating adipogenic differentiation, adipogenesis and fatty acid metabolism of hAMSCs. We also revealed that the underlying mechanism of MEK6-AS1 in regulating adipogenesis is by regulating the mRNA stability of its host gene *MEK6* through N-acetyltransferase 10 (NAT10). Our findings may provide new insights for the molecular mechanisms of adipogenic differentiation, and suggest that MEK6-AS1 might have potential in intervening in obesity and its related diseases, such as MAFLD.

## Methods

### Preparation of clinical adipose samples

A portion of the adipose tissue from patients undergoing liposuction was isolated to obtain primary hAMSCs. The isolation and culture procedures of hAMSCs were described in previous studies and hAMSCs at passage 3–5 were used for the following experiments [34, 35]. In brief, adipose tissue washed with sterile PBS solution (Servicebio, Wuhan, China) was centrifuged for 3–5 min at a speed of 800 × g. The resulting adipose tissue was then mixed uniformly with 0.2% sterile collagenase P (Sigma-Aldrich, St. Louis, MO, USA) at a volume ratio of 3:1 and placed in a shaker at 37 °C for approximately 30 min until no visible tissue clumps remain. The digested product was filtered by 100 μm sieves, washed twice with sterile PBS solution (Servicebio), and then centrifuged at 800 × g for 8–10 min. The final cell pellet was resuspended in Dulbecco’s modified Eagle’s medium (DMEM)/F12 (Gibco, Waltham, MA, USA) containing 100 U/mL penicillin (Gibco), 100 µg/mL streptomycin (Gibco), 2% fetal bovine serum (Gibco), 10^–9^ M insulin transferrin selenium (Gibco), 10^–8^ M dexamethasone (Sigma-Aldrich), 10^–4^ M ascorbic acid 2-phosphate (Sigma-Aldrich), 10 ng/mL epidermal growth factor (Sigma-Aldrich) and 10 ng/mL platelet-derived growth factor bb (Sigma-Aldrich), and then transferred to T75 flasks (Thermo Fisher Scientific, Waltham, MA, USA). All flasks were placed in humidified 37 °C incubator with 5% CO_2_. The culture medium was replaced every 2–3 days, and cells were passaged or cryopreserved at approximately 90% density.

Another portion of the adipose tissue was incubated with TRIzol reagent (Invitrogen, Waltham, MA, USA) to extract total RNA, which was subsequently used for analyzing the expression correlation of adipogenic-related marker genes and lncRNAs. All experiments followed the procedures approved by the Ethics Committee of the Chinese Academy of Medical Sciences and School of Basic Medicine Peking Union Medical College (SB2023048).

### Differentiation and staining of hAMSCs

hAMSCs at passage 3 − 5 were exposed to adipogenic differentiation medium, which consisted of high glucose-DMEM (Gibco) supplemented with 10% fetal bovine serum (Gibco), 0.5 mM isobutylmethylxanthine (Sigma-Aldrich), 1 µM dexamethasone (Sigma-Aldrich) and 1 mM ascorbic acid (Sigma-Aldrich). Adipogenic differentiation medium was changed every 3 days. It should be noted that all overexpression assays involved in this study used half the concentration of the above adipogenic differentiation medium to treat the cells. Oil Red O staining (Solarbio, Beijing, China) was used for the detection of lipid droplets according to our previous studies.

hAMSCs were placed into osteogenic differentiation medium, which consisted of high glucose-DMEM (Gibco) supplemented with 10% fetal bovine serum (Gibco), 10 mM β-glycerophosphate (Sigma-Aldrich), 0.5 mM ascorbic acid (Sigma-Aldrich), and 10 µM dexamethasone (Sigma-Aldrich). Osteogenic differentiation medium was changed every 3 days. It should be noted that we used half the concentration of the above osteogenic differentiation medium to treat the cells which were silenced of MEK6-AS1. Alizarin Red (Servicebio, Wuhan, China) staining was performed to detect matrix mineralization deposition during the later stage of osteogenesis according to our previous studies.

### Microarray analysis

Total RNA from hAMSCs undergo adipogenic differentiation on day 0 and day 3 was extracted by TRIzol reagent (Invitrogen). Processing, quality control and hybridization of samples were conducted by Cnkingbio Biotechnology (Beijing, China) with Affymetrix mRNA and lncRNA microarray chips. We focused on differentially expressed lncRNAs based on these criteria: fold change ≥ 2, expression value ≥ 3 and p-value < 0.05 (day 3 versus day 0).

### Culture and induction of hepatocyte organoids

iPSCs were purchased from Nuwacell (Anhui, China). We induced iPSCs to generate hepatic progenitor cells as directed by the STEMdiffTM Hepatocyte Differentiation Kit (STEMCELL Technologies, Vancouver, Canada). The process took approximately 14 days. Next, we induced hepatic progenitor cells into hepatocyte organoids as directed by the Human HepatiCultTM Organoid Kit (STEMCELL Technologies). The process took about 10–14 days for the first culture and about 5–7 days thereafter. When most of the organoids grew to about 200 µm (usually day 5 after culture), the growth medium was switched to the differentiation medium and continued for 4–7 days to form organoids with mature liver function. We added sodium-Palmitate (final concentration 250 µM) and sodium-Oleate (final concentration 500 µM) for induction of hepatic steatosis organoid on the day 5 of the differentiation medium, and treated for 5 days with fresh medium change every 24 h.

### RNA extraction and qRT-PCR

Total RNA was extracted from cultured cells or human adipose tissue by TRIzol reagent (Invitrogen) according to manufacturer’s instructions. Reverse transcription of RNA was performed by oligo (dT) primer (Takara, Otsu, Japan) and M-MLV reverse transcriptase (Takara). qRT-PCR was conducted with SYBR Green Master Mix (Yeasen, Shanghai, China) using the QuantStudio 3 (Applied Biosystems, Waltham, USA). The relative expressions of target lncRNA and genes were normalized to GAPDH expression and evaluated using the comparative threshold cycle (2^−ΔΔCt^) method. Additional file 2: Table S1 lists the primers used in this study.

### Protein extraction and western blotting

The cells were lysed with RIPA buffer containing 1 mM PMSF (Beyotime, Shanghai, China) on ice for 30 min and protein was qualified with a BCA kit (Beyotime). Western blotting was performed as previously described. Protein fractions were separated by 10% SDS-PAGE gels and transferred to 0.22 µM PVDF membranes (Millipore, Billerica, MA, USA). The membranes were blocked with 5% defatted milk and incubated with specific primary antibodies against CEBPA (1:1000, 2295S, CST, Beverly, MA, USA), PPARG (1:5000, 16643–1-AP, Proteintech, Wuhan, China), FABP4 (1:20000, 12802-1-AP, Proteintech), PLIN1(1:1000, 9349S, CST), MEK6(1:1000, 8550S, CST), GAPDH (1:10000, 10494-1-AP, Proteintech) overnight at 4 °C, and then incubated with HRP–conjugated secondary antibody (1:5000, Neobioscience, Shenzhen, China) at room temperature for 1 h. Immunodetection was visualized by a chemiluminescent ECL reagent (Yeasen). It should be emphasized that we implemented PVDF membrane stripping for proteins that could not be tailored for isolation by protein marker, such as GAPDH (36KD) and MEK6 (38KD). We incubated the antibody for MEK6 first, washed the PVDF membrane with eluent after chemiluminescent, and then incubated the antibody for GAPDH.

### Rapid amplification of cDNA ends (RACE)

Total RNA extracted from hAMSCs was used to determine the transcription start site and the size of the MEK6-AS1 transcript by RNA ligase-mediated RACE. 5′ RACE was performed by a 5′/3′RACE Kit (Roche, Basel, Switzerland) and 3′ RACE was performed by a 3′ full race core kit (Takara). Additional file 2: Table S1 lists the primers used in this study.

### Nuclear and cytosolic RNA extraction

The RNA distributed in nuclear and cytosolic fractions was extracted according to the manufacturer’s protocol of a NE-PER™ Nuclear and Cytoplasmic Extraction Reagent kit (Thermo Fisher Scientific). qRT-PCR was carried out to assess the relative proportion in nuclear and cytosolic fractions.

### RNA fluorescence in situ hybridization (FISH)

The hAMSCs were fixed in 4% formaldehyde (Servicebio) for 10 min at room temperature after washing with PBS solution. Then cells were permeabilized in PBS solution containing 0.5% Triton X-100 (Sigma-Aldrich) for 5 min at 4 °C and washed 3 times with PBS solution. FISH was conducted using an RNA FISH Kit (RiboBio, Guangzhou, China) as manufacturer’s protocol described. Briefly, anti-MEK6-AS1, anti-U6, or anti-18S FISH probes were incubated with the cells in the dark at 37 °C overnight. Then, the cells were stained with DAPI and imaged using a confocal microscope (Olympus, Tokyo, Japan).

### Small interfering RNA (siRNA) transfection and lentivirus infection

siRNAs were used to transient knockdown of MEK6-AS1, *MEK6* and *NAT10* via lipofectamine3000 (Invitrogen) according to the manufacturer’s recommendations. We used LV3-pGLV-H1-GFP-Puro lentiviral vectors for stable knockdown or overexpression of MEK6-AS1 or MEK6. hAMSCs were infected at a multiplicity of infection (MOI) of 10, and stable cell lines were established by puromycin (Yeasen) treatment. The synthesis of siRNAs and the package of lentiviral vectors were all from GenePharma (Suzhou, China). Additional file 2: Table S1 lists the primers used in this study.

### Detection of free fatty acids (FFAs)

The hAMSCs undergo adipogenic differentiation on day 6 were washed with cold PBS solution, collected and snap-frozen with liquid nitrogen. 39 types of FFAs assays were performed at CALIBRA (Hangzhou, China) [36–41]. Preprocessing method: Weigh an appropriate amount of cell sample, add the internal standard Nonadecanoic acid (C19:0) solution and 10% sulfuric acid–methanol solution, shake, react in an oven at 62 °C for a certain time, add saturated NaCl and n-hexane, shake, centrifuge, take the supernatant, and then re-solute and put it on the machine after nitrogen blowing. Chromatographic conditions: chromatographic column Rt-2560 capillary column (100 m*0.25 mm*0.2 μm); Split injection by 1 μL; Injection port temperature 235 °C; Ion source temperature 230 °C; The transmission line temperature is 240 °C. The initial temperature of programmed temperature rise is 60 °C; Hold, rise to 140 °C at 35 °C /min, rise to 230 °C at 3 °C/min, hold, rise to 240 °C at 4 °C/min, hold. The carrier gas is helium. MS condition: electric spray ionization (EI) source, SIM scanning module, electronic energy 70 eV. Statistical analysis [39, 40]: All statistical analyses were performed with R language (R version 3.4.1: http://www.cran.com; Rstudio version 1.4.1717 https://www.rstudio.com; mixOmics version 6.10.9 http://mixomics.org/; randomForest version 4.6–14 https://www.rdocumentation.org/packages/randomForest/versions/4.6–14). Significantly changed metabolites between case and control groups were found by parametric (student’s t-Test, ANOVA) or non-parametric (Wilcox’s rank test, Kruskal–Wallis, etc.) statistical methods.

### RNA pull-down assay

Full length of MEK6-AS1 or MEK6 was synthesized and cloned into the KpnI / SacI site of pBluescript II SK + . In vitro transcription and replication for RNA probe was prepared by Biotin RNA labeling mix (Roche), T7 (or T3) RNA Polymerase (Thermo Fisher Scientific) and RNA structure buffer. Total cellular protein was extracted from 1 × 10^7^ hAMSCs in 0.5 mL RIP buffer (150 mM KCl, 25 mM Tris pH7.4, 0.5% NP40, 1 mM PMSF, 100U RNase inhibitor, Protease Inhibitor Cocktail Tablets (Roche), then was mixed with biotinylated RNA to incubate overnight and was added pre-washed Streptavidin Dynabeads (Invitrogen) to capture RNA–protein complexes. Finally, the RNA pull-down captured proteins were separated by 8–12% SDS-PAGE with high resolution silver staining, and cut out the difference bands for mass spectrometry analysis.

### mRNA stability analysis

hAMSCs transfected with siRNAs were treated with 5 µg/mL Actinomycin D (Selleck, Houston, TX, USA), then total RNA was extracted at 0 h, 2 h, 4 h, 6 h and 8 h. mRNA level was normalized to *GAPDH* expression and was analyzed by reference samples (0 h).

### Heterotopic lipid formation in vivo

hAMSCs with lenti-MEK6-AS1, sh-MEK6-AS1 or the controls were induced for 3 days of adipogenic differentiation. As we mentioned earlier, it should be noted that hAMSCs with lenti-MEK6-AS1 were placed into half the concentration of the above adipogenic differentiation medium for 3 days. 4^10^6^ cells suspended in 100 µL DMEM medium were mixed with 150 µL Matrigel (Corning, NY, USA) on ice, and then injected subcutaneously into two symmetrical sites on the lower dorsal surface of 6-week-old BALB/c nu/nu female mice (n = 3 per group). After standard feeding or high-fat diet (HFD) feeding, the implants were obtained, fixed in 4% paraformaldehyde, embedded in paraffin and sectioned (3 µm thick) for hematoxylin and eosin staining (H&E) and immunohistochemical staining (IHC). All animal experiments were performed with approval of the Ethics Committee of the Chinese Academy of Medical Sciences and Peking Union Medical College (SB2023048).

### Statistical analysis

Data were analyzed by GraphPad Prism9 (GraphPad Software Inc., La Jolla, CA, USA). Statistical significance between two groups or among multiple groups was analyzed using Student’s t-tests (two-sided) and one-way analysis of variance (ANOVA), respectively. Statistically significant differences were considered as follows: ∗ P < 0.05, ∗  ∗ P < 0.01, ∗  ∗  ∗ P < 0.001, ∗  ∗  ∗  ∗ P < 0.0001, ns: not significant.

## Results

### Expression characterization of target lncRNA in human adipose tissue, and during adipogenic differentiation and hepatic steatosis organoid formation

**Fig. 1:**
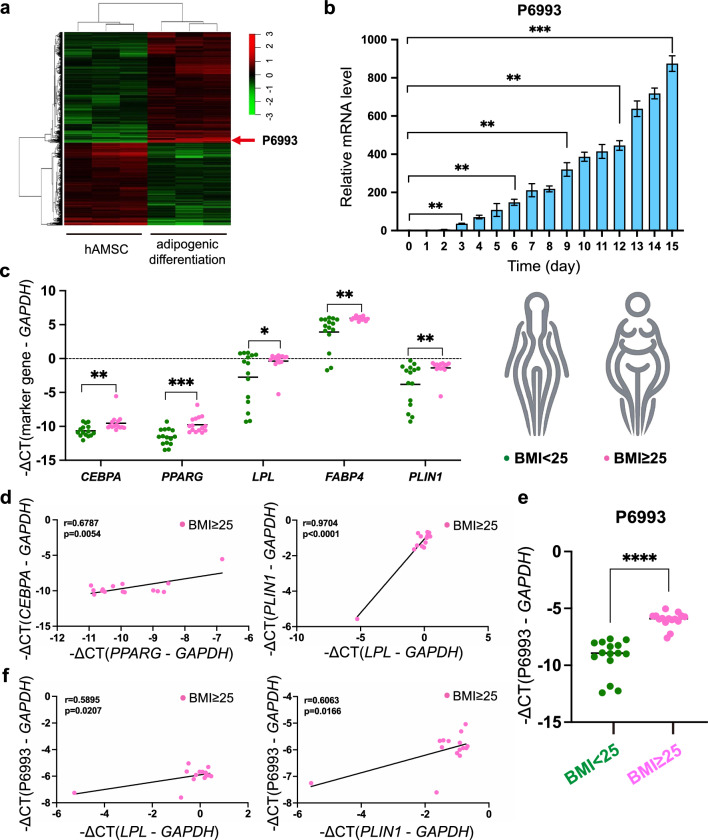
Expression characterization of target lncRNA P6993. **a** Heatmap of differentially expressed lncRNAs of normal and adipogenic-induced hAMSCs from three healthy donors. The red arrow indicated P6993 and the tentatively name was based on the lncRNA array probe. **b** qRT-PCR analysis was used to verify P6993 expression at different time points during adipogenic differentiation of hAMSCs. **c** The expression levels of key adipogenesis genes in human adipose tissue samples were detected by qRT-PCR. **d** Expression correlation between *CEBPA* and *PPARG* (left) and expression correlation between *PLIN1* and *LPL* (right) in BMI ≥ 25 samples were demonstrated. **e** The expression levels of P6993 in human adipose tissue samples were analyzed by qRT-PCR. **f** Expression correlation between P6993 and key adipogenesis genes in BMI ≥ 25 samples were demonstrated. p: p-value. r: correlation coefficient. When the absolute value of the correlation coefficient approaches 1, it indicates a stronger correlation between the variables. If the correlation coefficient is greater than 0, it indicates a positive correlation between the two. *GAPDH* was used as internal controls for qRT-PCR. The quantitative data were normalized to *GAPDH*, n = 3. Data are shown as the mean ± SD. Statistically significant differences were considered as follows: ∗P < 0.05, ∗  ∗P < 0.01, ∗  ∗  ∗P < 0.001

To identify human specific lncRNAs, we isolated MSCs from human adipose tissue (hAMSCs) and performed microarray analysis of lncRNAs after 3 days of adipogenic induction. hAMSCs were fibroblast-like and plastic-adherent cells (Additional file 1: Fig. S1a) and had multipotential to differentiate into adipocytes and osteocytes (Additional file 1: Fig. S1b, S1c). hAMSCs were positive for CD29, CD73, CD44, CD90, and CD105 expression and were negative for CD206, CD34, HLA-DR, CD45, and CD106 expression (Additional file 1: Fig. S1d) [42]. Combining stringent criteria (fold change ≥ 2, expression value ≥ 3, p-value < 0.05), we plotted a heat map of differentially expressed lncRNAs containing 35 up-regulated and 22 down-regulated lncRNAs (Fig. 1a). A lncRNA corresponding to probe p6993 (ENST00000585537.1, tentatively named as lncRNA P6993, P6993 for short) was the most significantly up-regulated, and we confirmed the dramatic elevation by qRT-PCR during 15 days adipogenic induction of hAMSCs (Fig. 1b).

We then used adipose tissues from human liposuction individuals to analyze the expression characteristics of lncRNA P6993. A total of 30 adipose tissue samples were collected from 15 adults with normal BMI (BMI < 25) and 15 adults with higher BMI (BMI ≥ 25). Firstly, we detected expression levels of key adipogenesis genes, such as *CEBPA, PPARG,* lipoprotein lipase (*LPL*)*,* fatty acid binding protein 4(*FABP4*) and perilipin 1 (*PLIN1*), which play critical roles in the development of obesity. Consistently, the expressions of these genes were relatively higher in BMI ≥ 25 human adipose tissue samples (Fig. 1c), and we also observed positive correlation in the expression of these genes in these samples (Fig. 1d). Next, we examined the expression levels of P6993 in the 30 human adipose tissue samples. Interestingly, the expression level of P6993 was significantly higher in BMI ≥ 25 samples than in the other group (Fig. 1e) and showed a closely positive correlation with key adipogenesis genes (Fig. 1f).

MAFLD is closely associated with obesity, and we also explored whether P6993 exhibits similar changes in it. We used induced pluripotent stem cells (iPSCs) as seeding cells to construct hepatic steatosis organoids to mimic MAFLD (Additional file 1: Fig. S2a, S2b). In this process, iPSCs were first induced into hepatic progenitor cells. Alpha fetoprotein (*AFP*) as a marker was elevated during this process (Additional file 1: Fig. S2c). Then, hepatic progenitor cells were induced into hepatocyte organoids in the growth medium. Cytochrome P450 family 3 subfamily A member 4 (*CYP3A4*) as an important marker was elevated during this process (Additional file 1: Fig. S2d). Hepatocyte organoids were induced into hepatic steatosis organoids in the differentiation medium supplemented with sodium-Palmitate and sodium-Oleate [43]. Compared with hepatocyte organoids, hepatic steatosis organoids had higher expression of the relevant markers such as perilipin 2 (*PLIN2*) and fatty acid binding protein 1(*FABP1*), which indicated the effective establishment (Additional file 1: Fig. S2e). Furthermore, P6993 as well as dramatically elevated in hepatic steatosis organoids (Additional file 1: Fig. S2e). The above results indicated that lncRNA P6993 may have significant implications in the homeostasis of human adipose tissue, and may be associated with the development of obesity-related diseases, such as MAFLD.

### Identification and characterization of lncRNA MEK6-AS1

**Fig. 2:**
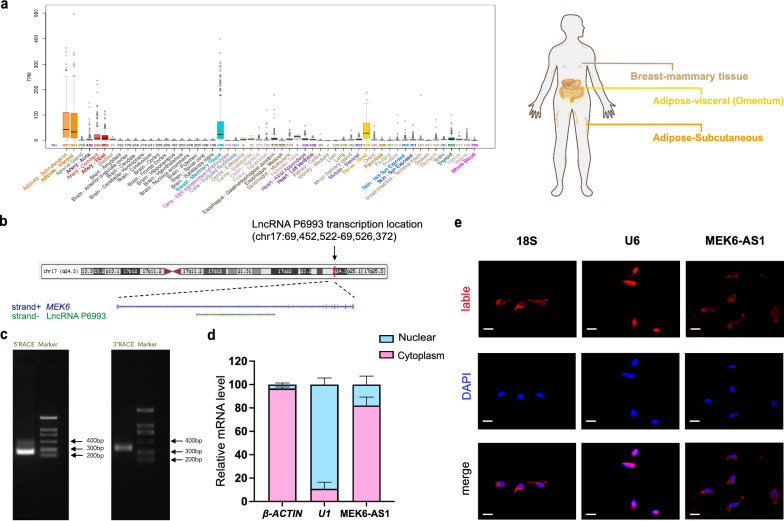
Identification and characterization of lncRNA MEK6-AS1. **a** Expression pattern of P6993 in human different tissues according to the UCSC Genome Browser. **b** Location of P6993 on human chromosome 17 according to the UCSC Genome Browser. **c** RACE assays were performed to validate the full length of MEK6-AS1. **d** MEK6-AS1 expression in fractionations of hAMSCs was detected by qRT-PCR. β-*ACTIN* mRNA served as the cytoplasmic control and *U1* mRNA served as the nuclear control. **e** Immunofluorescence images of the distribution of MEK6-AS1 in cells was analyzed by FISH assay. *18S* mRNA served as the cytoplasmic control and *U6* mRNA served as the nuclear control. Scale bar: 20 µm. *GAPDH* was used as internal control for qRT-PCR. The quantitative data were normalized to *GAPDH*, n = 3. Data are shown as the mean ± SD, n = 3

Prior to functional assays, we validated and characterized lncRNA P6993. Firstly, P6993 is specifically highly expressed in human adipose and breast tissues according to the UCSC Genome Browser (http://genome.ucsc.edu/, Fig. 2a). At a more microscopic level, P6993 is mapped to the intron1 related region of Mitogen-activated protein kinase kinase 6 (*Map2k6*, also known as *MEK6*) on human chromosome 17 (Fig. 2b) according to the UCSC Genome Browser. Under the generalized classification and naming rules for lncRNAs, we decided to designate P6993 (ENST00000585537.1) as lncRNA MEK6-AS1 (abbreviated as MEK6-AS1), which means it was located on the antisense strand of the intron1 of *MEK6*. The full length of MEK6-AS1 was 584nt by RACE assay (Fig. 2c, Additional file 1: Fig. S3). We found that MEK6-AS1 was mainly distributed in the cytoplasm after nuclear and plasmid separation of hAMSCs (Fig. 2d) and RNA FISH assay (Fig. 2e).

### Overexpression of MEK6-AS1 promotes the adipogenic differentiation of hAMSC in vitro, while knockdown of MEK6-AS1 inhibits this process

**Fig. 3:**
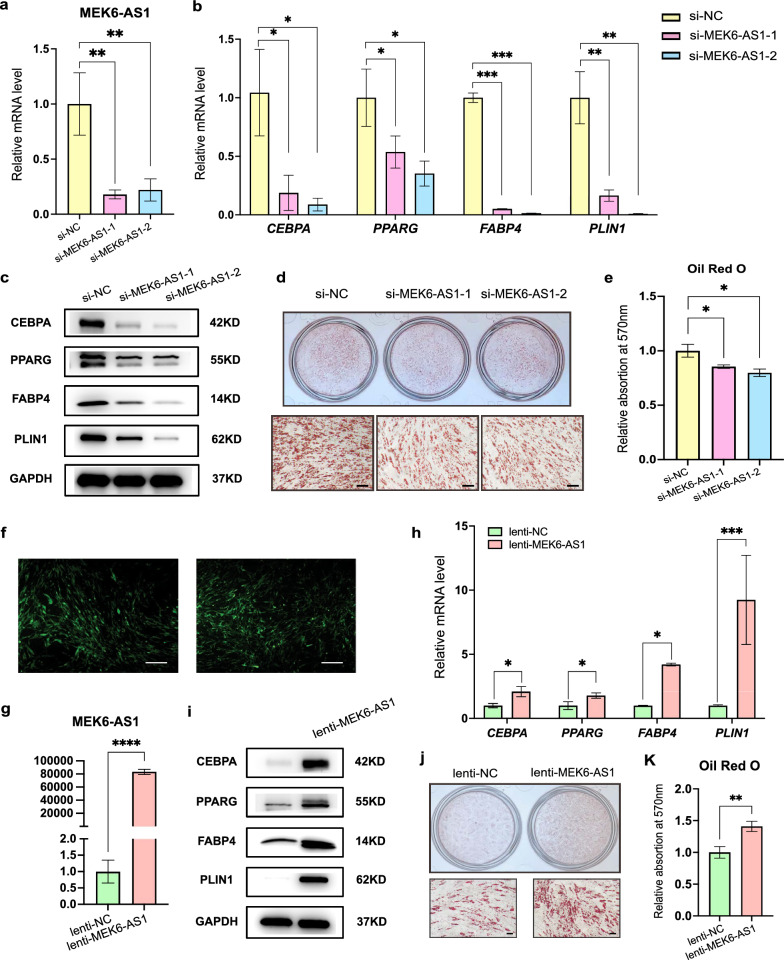
The effects of MEK6-AS1 on the adipogenic differentiation of hAMSCs. **a** MEK6-AS1 was silenced in hAMSCs using two independent pair of siRNAs (si-MEK6-AS1-1 and si-MEK6-AS1-2). The knockdown efficiency was verified by qRT-PCR as compared with the negative control (si-NC). **b** The expression of adipogenic markers in hAMSCs with MEK6-AS1 knockdown and in control hAMSCs on day 6 after adipogenic induction were analyzed by qRT-PCR. **c** Western blot analysis was performed to validate the expression of adipogenic markers in MEK6-AS1 knockdown hAMSCs or control hAMSCs on days 6 after adipogenic induction. **d** and **e** Oil Red O staining was used to confirmed the generation of lipids in cells treated with MEK6-AS1 siRNAs and corresponding control cells on day 10 after adipogenic induction (**d**) and the quantification of Oil Red O staining (**e**). Scale bars: 100 µm. **f** and **g** hAMSCs were transduced with lentivirus overexpressing MEK6-AS1 (lenti-MEK6-AS1) or control lentivirus (lenti-NC), and overexpression efficiency was detected by fluorescence observation (**f**) and qRT-PCR (**g**). Scale bars: 200 µm. (h and i) qRT-PCR (**h**) and western blot (**i**) analysis were performed to validate the expression of adipogenic markers in MEK6-AS1-overexpressed cells and corresponding control cells on day 6 after adipogenic induction. **j** and **k** The gerenation of lipids in cells on day 10 after adipogenic induction were indicated by Oil Red O staining (**j**) and quantification of the Oil Red O staining (**k**). Scale bars: 100 µm. *GAPDH* was used as internal control for qRT-PCR and western blot. The quantitative data were normalized to *GAPDH*, n = 3. Data are shown as the mean ± SD. Statistically significant differences were considered as follows: ∗P < 0.05, ∗  ∗P < 0.01, ∗  ∗  ∗P < 0.001, ∗  ∗  ∗  ∗P < 0.0001

To investigate the function of MEK6-AS1, we next used two pairs of specific siRNAs to inhibit the endogenous expression of MEK6-AS1 (Fig. 3a), and then hAMSCs were induced for adipogenic differentiation. qRT-PCR and western blot confirmed that several adipogenic-related markers such as CEBPA, PPARG, PLIN1 and FABP4 were significantly down-regulated at both mRNA and protein levels (Fig. 3b, c). Consistently, Oil Red O staining also revealed a reduction in lipid droplet generation after MEK6-AS1 knockdown (Fig. 3d), and quantitative analysis after extraction showed a similar tendency (Fig. 3e).

Then, we used green fluorescent protein (GFP)-tagged lentivirus to stably express full length MEK6-AS1 in hAMSCs (Fig. 3f, g). qRT-PCR and western blot showed that hAMSCs overexpressing MEK6-AS1 (lenti-MEK6-AS1) had a significantly higher adipogenic differentiation capacity compared to empty vector-infected hAMSCs (lenti-NC) (Fig. 3h, i). Additionally, Oil Red O staining assay indicated that overexpression of MEK6-AS1 markedly enhanced the efficiency of adipogenic differentiation (Fig. 3j, k).

Moreover, obesity is closely associated with inflammation, and MSCs are characterized by the secretion of various cytokines [42, 44]. Therefore, we performed ELISA assays to detect the levels of several inflammatory cytokines in the culture supernatants of cells after 6 days of adipogenic induction, following the knockdown or overexpression of MEK6-AS1. The results demonstrated that during the process of adipogenic differentiation, the expression levels of pro-inflammatory cytokines such as Interleukin-1beta (IL-1beta), Interleukin-6 (IL-6), Monocyte chemoattractant protein-1 (MCP-1) were significantly increased or decreased following the knockdown or overexpression of MEK6-AS1 (Additional file 1: Fig. S4).

### MEK6-AS1 modulates fatty acid metabolism during adipogenic differentiation of hAMSCs

**Fig. 4:**
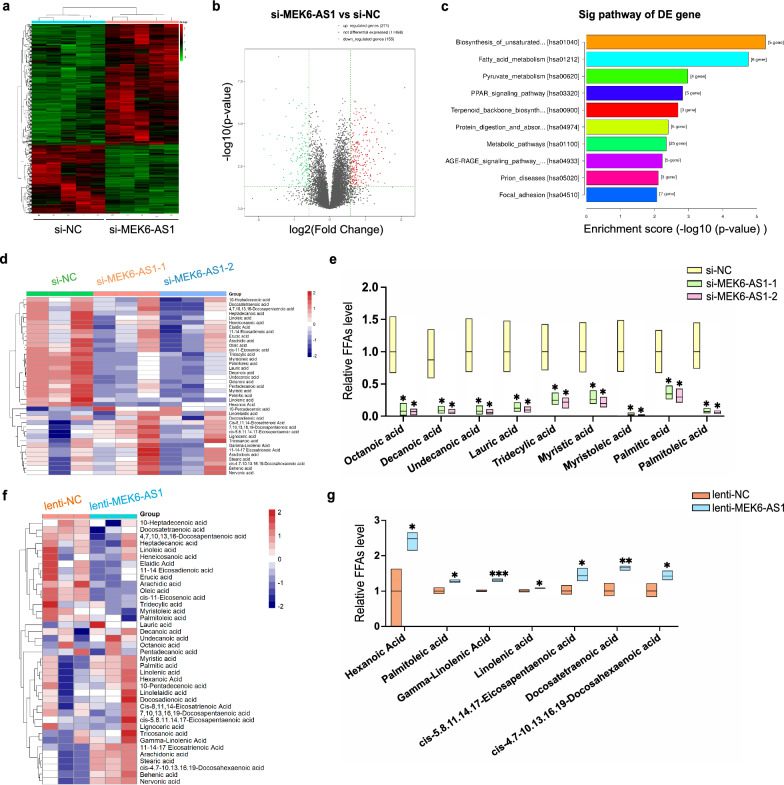
Effect of MEK6-AS1 on fatty acid metabolism during the differentiation of MSCs into adipocytes. **a** Heatmap of differentially expressed genes between MEK6-AS1 knockdown and control cells. n = 5. **b** Volcano mapping of differentially expressed genes (fold change ≥ 1.5 and p-value ≤ 0.5). A total of 271 up-regulated genes and 155 down-regulated genes were detected. **c** KEGG pathway enrichment analysis revealed that the 155 down-regulated genes were mainly enriched in fatty acid metabolism related pathways. **d and f** Hierarchical clustered heatmap of the concentrations of 39 types of FFAs in cells with MEK6-AS1 knockdown (**d**) or overexpress (**f**) and in control cells. Each color block represented the concentration value of each FFA in each sample, and the values pre-standardized beforehand. n = 3. **e** All significantly down-regulated FFAs in adipogenic-induced cells after silence of si-MEK6-AS1-1 and si-MEK6-AS1-2. **g** Mass spectrometry showed that the relative contents of all significantly up-regulated FFAs in adipogenic-induced cells after overexpression of MEK6-AS1. Data are shown as the mean ± SD. Statistically significant differences were considered as follows: ∗P < 0.05, ∗  ∗P < 0.01, ∗  ∗  ∗P < 0.001

To explore the dramatic effect of MEK6-AS1 on the process of fatty acid metabolism, we also performed RNA transcriptome sequencing of MEK6-AS1 silenced cells and their control cells after adipogenic induction for 3 days. By cluster analysis (Fig. 4a) and volcano mapping (Fig. 4b) of differentially expressed genes (fold change ≥ 1.5 and p-value ≤ 0.5), a total of 271 up-regulated genes and 155 down-regulated genes were detected. KEGG pathway enrichment analysis revealed that the down-regulated genes were mainly enriched in fatty acid metabolism related pathways, such as biosynthesis of unsaturated fatty acids signaling pathway, fatty acid metabolism signaling pathway and PPAR signaling pathway (Fig. 4c). We also validated several key down-regulated genes such as fatty acid desaturase 1 (*FADS1*)*,* stearoyl-CoA desaturase (*SCD*), ELOVL fatty acid elongase 6 (*ELOVL6*) and acetyl-CoA carboxylase alpha (*ACACA*) involved in these pathways by qRT-PCR (Additional file 1: Fig. S4a). We also detected a decrease in the expression of genes involved in the process of fatty acid esterification and lipid droplets formation, including Acyl-CoA synthetase long chain family member 3 (*ACSL3*), glycerol-3-phosphate acyltransferase 3 (*GPAT3*), 1-acylglycerol-3-phosphate O-acyltransferase 2 (*AGPAT2*), and Acyl CoA: Diacylgycerol acyltransferase 2 (*DGAT2*) after the knockdown of MEK6-AS1 (Additional file 1: Fig. S5a). In order to further explore the regulation of MEK6-AS1 on these fatty acid metabolic pathways, we compared the level of 39 types of free fatty acids (FFAs) in MEK6-AS1 knockdown or overexpression cells and their corresponding control cells by targeted metabolomics. The results of mass spectrometry showed that the relative contents of Octanoic acid, Decanoic acid, Undecanoic acid, etc. decreased significantly after knockdown of MEK6-AS1 (Fig. 4d, e, Additional file 1: Fig. S5b), and the relative contents of Palmitoleic acid, Linolenic acid, etc. increased obviously after overexpression of MEK6-AS1 (Fig. 4f, g, Additional file 1: Fig. S5c). These results suggest that MEK6-AS1 can significantly modulate the fatty acid metabolism during the differentiation of hAMSCs into adipocytes.

In addition, we also analyzed the 271 up-regulated genes after MEK6-AS1 knockdown. Interestingly, we found multiple genes related to bone development, such as collagen type XI alpha 1 chain (*COL11A1*), 15-hydroxyprostaglandin dehydrogenase (*HPGD*) and frizzled related protein (*FRZB*), were obviously elevated. As adipogenic differentiation and osteogenic differentiation of MSCs is closely related, we then induced osteogenic differentiation of hAMSCs after knockdown or overexpression of MEK6-AS1 [45, 46]. The results showed that the expression of osteogenic marker genes such as RUNX family transcription factor 2 (*RUNX2*), alkaline phosphatase (*ALP*), secreted phosphoprotein 1 (*OPN*), collagen type I alpha 1 chain (*COL1A1*) were up-regulated (Additional file 1: Fig. S6a) and matrix mineralization deposition was increased (Additional file 1: Fig. S6b) after knockdown of MEK6-AS1, while overexpression of MEK6-AS1 decreased these indicators (Additional file 1: Fig. S6c, S6d). We speculated that MEK6-AS1 may play a critical role in promoting adipogenic differentiation and inhibiting osteogenic differentiation for human MSCs. Taken together, we demonstrated that MEK6-AS1 had a prominent regulatory effect on fatty acid metabolism of hAMSCs during adipogenic differentiation in vitro.

### MEK6-AS1 enhances adipogenesis of hAMSCs in vivo

**Fig. 5:**
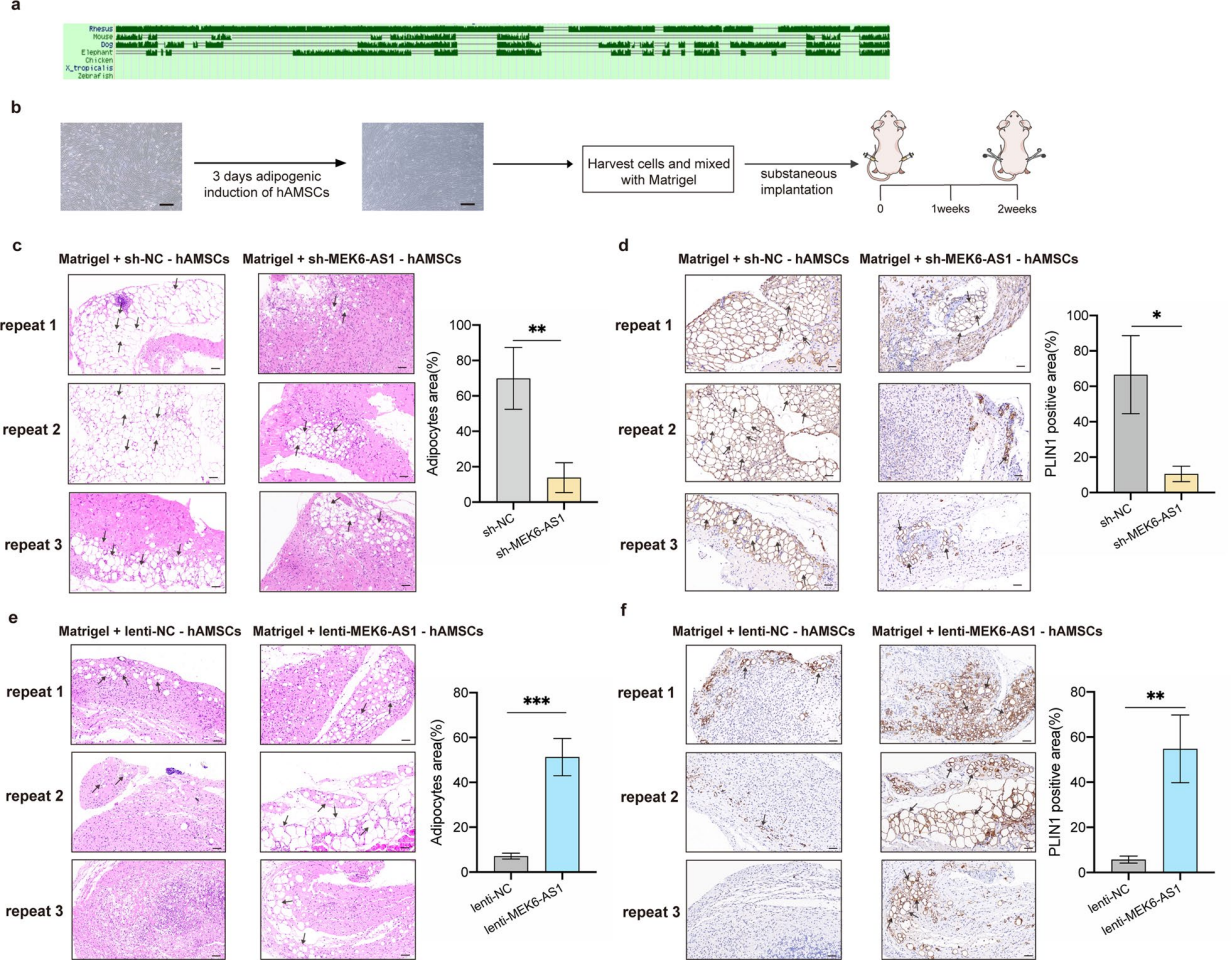
MEK6-AS1 enhances adipogenesis of hAMSCs in vivo. **a** The conservatism of MEK6-AS1 is demonstrated according to the UCSC Genome Browser. **b** Schematic of the in vivo experimental setup. hAMSCs were infected with lentivirus (lenti-NC, lenti-MEK6-AS1, sh-NC, sh-MEK6-AS1) and induced to undergo adipogenic differentiation for 3 days. Cells were harvested and mixed with Matrigel on ice and then were subcutaneously injected into 6-week-old BALB/c nu/nu mice. The adipose plugs were collected 2 weeks later for analysis. Scale bars: 200 µm. **c** and **d** Representative H&E staining (**c**) and IHC staining (**d**) images for de novo adipogenesis in BALB/c nu/nu mice injected with MEK6-AS1 overexpression and control cells. n = 3. Scale bars: 50 µm. Black arrows indicated adipocytes formed by differentiation of hAMSCs. **e** and **f** Representative H&E staining (**e**) and IHC staining (**f**) assays were performed to validate the de novo adipogenesis in BALB/c nu/nu mice injected with MEK6-AS1 knockdown and control cells. n = 3. Scale bars: 50 µm. Black arrows indicated adipocytes formed by differentiation of hAMSCs

The UCSC Genome Browser showed MEK6-AS1 (ENST00000585537.1) exhibited relatively low conservation and only exists in human, and high abundance expression mainly in human adipose and breast tissues (Fig. 2a). In addition, MEK6-AS1 was not conserved in model animals such as mice, which made it less feasible to establish gene editing-based disease models (Fig. 5a). Therefore, to further validate the functional role of MEK6-AS1 on adipogenesis, we conducted ectopic lipid formation experiments in nude mice.

hAMSCs infected with specific lentivirus (lenti-NC, lenti-MEK6-AS1, sh-NC, sh-MEK6-AS1) to stably overexpress or knockdown MEK6-AS1 were placed into adipogenic differentiation medium for 3 days. 6-week-old BALB/c nu/nu mice (n = 3) were injected subcutaneously with pre-induced cells (lenti-NC, lenti-MEK6-AS1) mixed with Matrigel into two symmetrical sites on the lower dorsal surface. PLIN1 is a major lipid droplet-binding protein and serves as a specific important marker of adipogenesis. These implants were obtained for H&E staining and PLIN1-targeted IHC staining after 2 weeks (Fig. 5b). Another group of mice (n = 3) underwent the same process, while injected with pre-induced hAMSCs transfected with sh-NC or sh-MEK6-AS1. H&E staining showed that the efficiency of differentiation towards adipocytes was dramatically decreased in sh-MEK6-AS1 group (Fig. 5c). As an assessment marker, expression of PLIN1 was also significantly down-regulated (Fig. 5d). While the overexpression of MEK6-AS1 enhanced lipid formation of hAMSCs, as evidenced by the higher efficiency of differentiation towards adipocytes and expression of PLIN1 in sh-MEK6-AS1 group (Fig. 5e, f).

Additionally, we investigated the effect of MEK6-AS1 on adipogenesis in vivo under under HFD feeding conditions. The treatment for all transplanted cells remained consistent, with all mice being fed a high-fat diet following subcutaneous cell injection. The results indicated that the adipogenic differentiation capacity of cells in the knockdown group was significantly reduced, accompanied by a lower expression level of PLIN1 (Additional file 1: Fig. S7a, S7b). Conversely, the adipogenic differentiation capacity of cells in the overexpression group was significantly enhanced, with an increased expression level of PLIN1 (Additional file 1: Fig. S7c, S7d). These findings suggested that under HFD conditions, the knockdown or overexpression of MEK6-AS1 continues to exert inhibitory or promotive effects, respectively, on the differentiation of hAMSCs into adipocytes in vivo.

Accordingly, in vivo experiments we also assessed the levels of inflammatory cytokines. Under standard feeding conditions, IHC staining indicated that knockdown or overexpression of MEK6-AS1 led to a decrease or increase in the secretion levels of IL-1beta and MCP-1 during adipogenesis (Additional file 1: Fig. S8a, S8b). Furthermore, under HFD feeding conditions, MEK6-AS1 similarly exerted a significant influence on the expression of IL-1beta and MCP-1 (Additional file 1: Fig. S8c, S8d).

### The host gene *MEK6* has similar role with MEK6-AS1 in the regulation of adipogenic differentiation

**Fig. 6:**
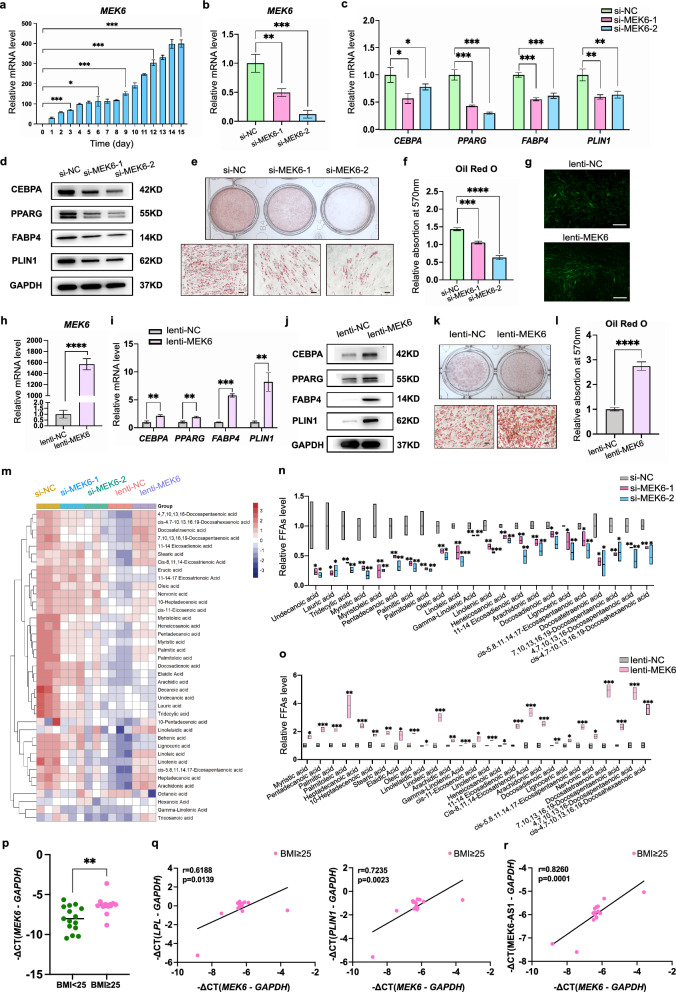
Effects of MEK6 on adipogenic differentiation and fatty acid metabolism of hAMSCs. **a** qRT-PCR was used to verify *MEK6* expression at indicated time points during adipogenic differentiation of hAMSCs. **b** *MEK6* was silenced using two pairs of siRNAs. **c** and **d** qRT-PCR (**c**) and western blot (**d**) were performed to analyze adipogenic markers in each group of cells on day 6 after adipogenic induction. **e** and **f** Oil Red O staining and quantification were used to verify generation of lipids in each group of cells on day 10 after adipogenic induction. Scale bars: 100 µm. **g** and **h** hAMSCs were transduced with lentivirus and overexpression efficiency was detected by fluorescence observation (**g**) and qRT-PCR (**h**). Scale bars: 200 µm. **i** and **j** qRT-PCR (**i**) and western blot (**j**) were used to analyze expression of adipogenic markers in cells on day 6 after adipogenic induction. **k** and **l** Generation of lipids in cells on day 10 after adipogenic induction were detected by Oil Red O staining (**k**) and quantification of Oil Red O staining (**l**). Scale bars: 100 µm. **m** Hierarchical clustered heatmap of concentrations of 39 types of FFAs in each group of cells. Each color block represented the concentration value of each FFA in each sample, and the values pre-standardized beforehand. n = 3. **n** and **o** Mass spectrometry showed that the relative contents of all significantly down-regulated FFAs in cells with *MEK6* knockdown (**N**) or overexpress (**O**) and in control cells. **p** Expression levels of *MEK6* in BMI < 25 and BMI ≥ 25 adipose tissue samples by qRT-PCR. **q** Expression correlation between *LPL* or *PLIN1* and *MEK6* in BMI ≥ 25 samples. **r** Expression correlation between MEK6-AS1 and *MEK6* in BMI ≥ 25 samples.*GAPDH* was used as internal controls for qRT-PCR and western blot. The quantitative data were normalized to *GAPDH*, n = 3. Data are shown as the mean ± SD. Statistically significant differences were considered as follows: ∗P < 0.05, ∗  ∗P < 0.01, ∗  ∗  ∗P < 0.001, ∗  ∗  ∗  ∗P < 0.0001

Next, we further explored how MEK6-AS1 regulated the process of adipogenic differentiation of hAMSCs. As we mentioned above, MEK6-AS1 is located in the intron1 related region of *MEK6* in human genome (Fig. 2b). It's worth noting that *MEK6* had been reported to be associated with white adipose tissue and obesity in mice. We postulate that *MEK6* may be involved in the regulation of adipose differentiation by MEK6-AS1. We initially detected that the expression level of *MEK6* was also dramatically increased during adipogenic differentiation of hAMSCs (Fig. 6a). Then, through loss- and gain-of-function assays, we revealed that *MEK6* had strong effect on adipogenic differentiation of hAMSCs. In brief, knockdown of endogenous *MEK6* using two pairs of specific siRNAs (Fig. 6b) showed a dramatically decrease in adipogenic differentiation-related markers (Fig. 6c, d), lipid droplets (Fig. 6e, f) and FFAs levels such as Lauric acid, Tridecylic acid, Myristic acid, and Docosatetraenoic acid (Fig. 6m, n, Additional file 1: Fig. S9a). Overexpression of *MEK6* by GFP-tagged lentivirus (Fig. 6g, h) demonstrated enhanced increase of the same indicators (Fig. 6i, j, k, l, m, n and o, Additional file 1: Fig. S9b). Notably, *MEK6* was also highly expressed in BMI ≥ 25 human adipose tissue samples (Fig. 6p) and had positive expression correlation with key adipogenesis genes and MEK6-AS1 (Fig. 6q, r). *MEK6* has similar regulatory effects as MEK6-AS1, suggesting that *MEK6* may be associated with the regulation of MEK6-AS1 on adipogenic differentiation and fatty acid metabolism.

### MEK6-AS1 potentially modulates the mRNA stability of *MEK6* by interacting with NAT10

**Figure.7:**
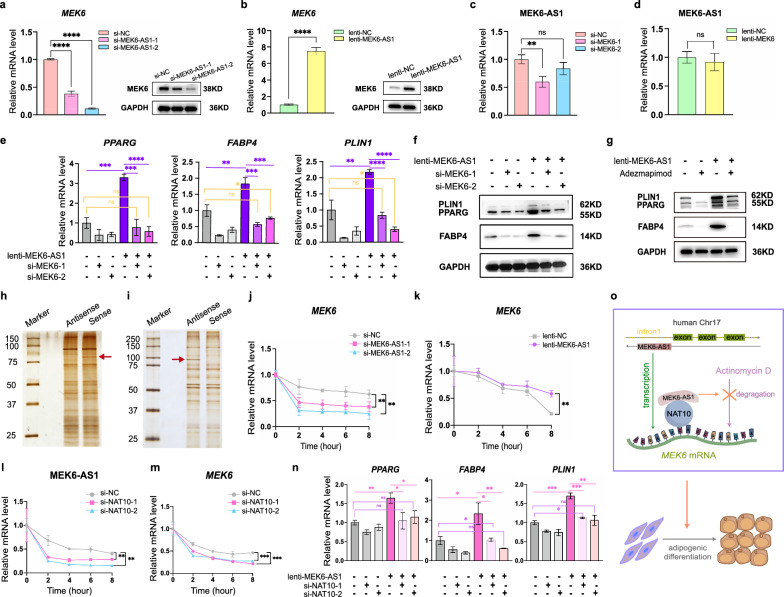
MEK6-AS1 potentially modulates mRNA stability of MEK6. **a** qRT-PCR and western blot were used to verify *MEK6* expression in cells with MEK6-AS1 knockdown and control group during adipogenic differentiation. It should be emphasized that the GAPDH band is same to Fig. 3C. **b** qRT-PCR and western blot were used to verify MEK6 expression of cells infected with MEK6-AS1 overexpression virus or control virus during adipogenic differentiation. It should be emphasized that the GAPDH band is same to Fig. 3I. **c and d** qRT-PCR analyzed expression of MEK6-AS1 in hAMSCs after knockdown (**c**) or overexpression (**d**) of *MEK6*. **e** and **f** qRT-PCR (**e**) and western blotting (**f**) detected adipogenic markers in cells after *MEK6* knockdown followed by MEK6-AS1 overexpression. **g** Western blotting detected adipogenic markers in cells with Adezmapimod (a MAPK pathway inhibitor) followed by MEK6-AS1 overexpression. **h** and **i** Silver staining showed that there was one differential band each between sense and anti-sense of MEK6-AS1 (**h**) and *MEK6* mRNA (**i**). The red arrows indicated differential bands.(j and k) qRT-PCR of MEK6 stability in the presence of MEK6-AS1 knockdown (**j**) and overexpression (**k**) in cells treated with Actinomycin D for 0 h, 2 h, 4 h, 6 h and 8 h. **l** and **m** qRT-PCR of MEK6-AS1 stability (l) and *MEK6* stability **m** in *NAT10* silenced cells and control cells treated with Actinomycin D for 0 h, 2 h, 4 h, 6 h and 8 h. **n** qRT-PCR detected adipogenic marker genes in cells after *NAT10* knockdown followed by MEK6-AS1 overexpression. **o** Schematic representation of the mechanism that MEK6-AS1 promotes adipogenic differentiation of hAMSCs by regulating *MEK6* mRNA stability. *GAPDH* was used as internal control for qRT-PCR and western blot. The quantitative data were normalized to *GAPDH*. n = 3. Data are shown as the mean ± SD. Statistically significant differences were considered as follows: ∗P < 0.05, ∗  ∗P < 0.01, ∗  ∗  ∗P < 0.001, ∗  ∗  ∗  ∗P < 0.0001, ns: not significant

To further elucidate whether MEK6-AS1 affects adipogenic differentiation by regulating its host gene *MEK6*, we explored the correlation between MEK6-AS1 and *MEK6* during adipogenic differentiation of hAMSCs. We noticed that the expression of MEK6 was suppressed after knockdown of MEK6-AS1 (Fig. 7a) and increased after overexpression of MEK6-AS1 (Fig. 7b). However, the expression of MEK6-AS1 was suppressed after knockdown of *MEK6* while there was no significant change in the expression of MEK6-AS1 after overexpression of *MEK6* (Fig. 7c, d). Furthermore, the positive regulation on adipogenic differentiation of hAMSCs by overexpressing of MEK6-AS1 was significantly impaired by knockdown of *MEK6*, as indicated by dramatic down-regulation of adipogenic-related markers at both mRNA and protein levels (Fig. 7e, f). Since the MEK protein family acts as an upstream agonist of the MAPK signaling pathway, we also used MAPK pathway inhibitor (Adezmapimod, Selleck) to further investigate the effects of *MEK6* on adipogenic differentiation of hAMSC. We found that adipogenic differentiation of hAMSCs was inhibited by Adezmapimod upon overexpression of MEK6-AS1, as indicated by decrease of PPARG, FABP4 and PLIN1 (Fig. 7g). We thus inferred that MEK6-AS1 improved the adipogenic differentiation of hAMSCs at least partially by affecting *MEK6*.

We firstly revealed that MEK6-AS1 could not bind directly to *MEK6* by RNA Binding Protein Immunoprecipitation (RIP) experiment (Additional file 1: Fig. S10a). We performed RNA Pull Down assays on MEK6-AS1 and mRNA of *MEK6* separately. The results showed that there was one differential band each between sense and anti-sense of MEK6-AS1 and *MEK6* mRNA (Fig. 7h, i). After mass spectrometry detection of the two bands individually, we found that MEK6-AS1 might bind 207 proteins and *MEK6* mRNA might bind 249 proteins. A total of 31 proteins in the two groups were overlapped (Additional file 1: Fig. S10b). Among them, NAT10, which is known for promoting mRNA stability, strongly attracted our attention. We went on to analyze the underlying mechanism through Actinomycin D-mediated mRNA stability assays. Firstly, knockdown of MEK6-AS1 resulted in more degradation of *MEK6* mRNA (Fig. 7j), whereas overexpression of MEK6-AS1 reduced degradation of *MEK6* mRNA (Fig. 7k). We also silenced *NAT10* through two pairs of siRNAs, then the degradation of MEK6-AS1 and *MEK6* were both increased in such a case (Fig. 7l, m). Additionally, the positive regulation on adipogenic differentiation of hAMSCs by overexpression of MEK6-AS1 was also impaired by knockdown of *NAT10*, as indicated by declination of *PPARG*, *FABP4* and *PLIN1* (Fig. 7n). Collectively, these data suggested that MEK6-AS1 could potentially modulates the mRNA stability of *MEK6* by NAT10 (Fig. 7o).

## Discussion

One of the most distinctive features of obesity is 'heterogeneity' [47, 48], which refers to the differences between obese patients in a number of aspects, including pathogenesis, symptomatology and response to treatment. Heterogeneity further increases the uncertainty of responses to weight-loss drugs and makes obesity a complex and multifaceted health issue [49–51]. Multiple factors can contribute to obesity heterogeneity [52–55], such as hormone levels [56–58], environments [59, 60] and diet structure [61, 62]. In fact, genetic and epigenetic factors play a vital role on heterogeneity, because variations in individual genes may lead to differences in energy metabolism and appetite control [63–66]. In recent years, lncRNAs have been found to play an important role in many physiological and pathological processes as an epigenetic regulatory mediator [67–71]. Although several lncRNAs have been found to be associated with adipogenesis and lipid metabolism in mice or cell lines [72–74]. Due to the poor conservation of lncRNAs, functional lncRNAs found in one species may not exist in other species, which greatly reduces the value of functional lncRNAs found in animals for human-related diseases [75, 76]. We have discovered a lncRNA whose function has not yet been reported. The UCSC Genome Browser showed that this lncRNA was an antisense lncRNA of *MEK6*, known as MEK6-AS1 (ENST00000585537.1). MEK6-AS1 exhibits relatively low conservation and only exists in human and is not conserved in model animals such as mice, zebrafish, rabbit. Although the non-conservative nature of MEK6-AS1 makes it difficult to indirectly investigate its function in humans by constructing animal disease models, the verification of its function and mechanism are more instructive for the diagnosis and treatment of human-related diseases.

The low conserved MEK6-AS1 has rarely been reported in previous studies. We found that MEK6-AS1 was mainly highly expressed in human adipose and breast tissues, and the expression level of MEK6-AS1 was significantly increased during adipogenic differentiation. These expression features strongly suggest that MEK6-AS1 may play a key role in adipogenesis. JianJiang Zhao et al. found a decrease of ENST00000585537.1 during osteogenic differentiation of MSCs derived from human bone marrow [77]. This work also suggested us that MEK6-AS1 might be important to adipogenic differentiation of MSCs. Furthermore, by loss- and gain-of-function assays we demonstrated that MEK6-AS1 had a positive regulatory effect on adipogenic differentiation of hAMSCs in vitro and on adipogenesis in vivo, which was corroborated to some extent by its high abundance in human subcutaneous adipose tissue, visceral adipose tissue and breast tissue (rich in adipose tissue). We then verified that the expression level of MEK6-AS1 was positively correlated with the expression of genes related to adipogenesis in human adipose tissues with high BMI, which further highlighting the significance of MEK6-AS1 for human adipose tissue homeostasis and overweight or obesity. In addition, we explored the genes which were significantly up-regulated after knockdown of MEK6-AS1, and found that multiple genes were associated with osteogenesis-induced differentiation. This was also in line with the work of JianJiang Zhao et al. and further illustrated the fine regulatory role of MEK6-AS1 on hAMSCs differentiation. As mentioned above, MEK6-AS1 is poorly conserved in different species, it is difficult to generate conditional knockout mice to investigate the role of MEK6-AS1 in vivo. Next, we used an in vivo model of ectopic adipogenesis to further validate the function of MEK6-AS1 under standard feeding or HFD feeding, this is a relatively feasible method to test the ability of differently treated hAMSCs to differentiate into adipocytes in vivo [78, 79]. There is no doubt that, MEK6-AS1 can significantly accelerate the adipogenesis of MSCs in vivo, consistent with the in vitro results of this process. In this study, in addition to focusing on the classic adipogenic differentiation phenotype, we also investigated the changes in inflammatory factors during this process through both in vivo and in vitro experiments. Obesity is closely associated with chronic inflammation, and the regulation of pro-inflammatory factors by MEK6-AS1 further enhanced its biological significance.

MAFLD is defined as hepatic steatosis that occurs in the absence of heavy alcohol consumption or other etiologies of chronic liver disease [80, 81]. According to epidemiological surveys, the healthcare burden associated with MAFLD exhibits a degree of synchronization with the rise in obesity rates [82]. About two-thirds of overweight and obese people are found to have MAFLD [83]. It indicates that overweight and obesity are important risk factors for MAFLD, and excessive intrahepatic adipocyte deposition ultimately leads to lipotoxicity and hepatocellular injury. We used iPSCs as seeding cells to construct hepatocyte organoids and hepatic steatosis organoids with the aim of exploring whether MEK6-AS1 changes during this complex process. We found no significant difference in the expression of MEK6-AS1 in the stages from iPSCs to hepatic progenitor cells and then to hepatocyte organoids. We speculated that this may be related to the specific expression of MEK6-AS1, which is mainly highly expressed in human adipose and mammary tissues compared to liver as we mentioned previously. However, at the stage when hepatocyte organoids were induced into hepatic steatosis organoids by FFAs loading, a significant rise of MEK6-AS1 occurred. These results confirmed the regulatory effect of MEK6-AS1 on obesity and its related pathological processes again, and further suggested that MEK6-AS1 may have an impact on fatty acid metabolism. Our results of RNA transcriptome sequencing combined with mass spectrometry analysis showed that MEK6-AS1 significantly affected fatty acid metabolism, which was manifested by the relative contents of Octanoic acid, Decanoic acid, Undecanoic acid, etc. The relative contents of Palmitoleic acid, Linolenic acid, etc. were significantly decreased after knockdown of MEK6-AS1 and increased after overexpression of MEK6-AS1. These data confirmed that MEK6-AS1 is indeed able to regulate fatty acid metabolism during the differentiation of hAMSCs into adipocytes.

Antisense lncRNAs currently account for 20% of total human and murine lncRNAs. Their distribution is not random, but tend to be inversely aligned and transcribed with genes encoding transcription factors and development-related genes. Antisense lncRNAs can cis-regulate the transcription of nearby protein-coding genes to finely control the spatiotemporal expression of these genes and participate in associated biological processes. MEK6-AS1 was located in the antisense strand of the first intron of *MEK6*, we thus focused on *MEK6* to explore the mechanism of MEK6-AS1 on adipogenic differentiation. The MEK protein family acts as an upstream agonist of the MAPK signaling pathway, whereas the association of *MEK6* with adipose tissue and obesity has also been reported [84]. For example, MEK6 was found to be highly expressed in epididymal and subcutaneous white adipose tissue of obese mice [85], and the differentiation of mouse preadipocytes could be inhibited by suppressing MEK6 [86]. However, there was no study reporting the role of MEK6 in the homeostasis of human adipose tissue and obesity so far. Through systematically studying the expression pattern of *MEK6* and its effect on adipogenic differentiation, we found that *MEK6* and MEK6-AS1 have similar expression patterns and similar regulatory functions. *MEK6* expression is up-regulated during adipogenic differentiation, and expression level of *MEK6* in human adipose tissue is positively correlated with the expression of key genes of adipogenesis. Overexpression of *MEK6* can significantly promote adipogenic differentiation and modulate FFAs levels. Furthermore, adipogenic differentiation of hAMSCs was inhibited by Adezmapimod (MAPK pathway inhibitor) upon overexpression of MEK6-AS1. These results suggest that *MEK6* may be directly or indirectly involved in the regulation of MEK6-AS1 for adipogenic differentiation and fatty acid metabolism.

Antisense lncRNAs have various regulatory mechanisms, such as serving as ceRNA, interacting with promoters, altering mRNA half-life, and regulating transcriptional splicing [87, 88]. MEK6-AS1 was mainly distributed in the cytoplasm, we then focus on exploring its potential regulatory mechanism at the post-transcriptional level. It has been reported that silencing 75% of randomly selected antisense lncRNAs led to a decrease in the expression of neighboring protein-coding genes [89]. In this study, we demonstrated that the expression of *MEK6* was also significantly decreased when MEK6-AS1 was knocked down, while the expression of *MEK6* was up-regulated after MEK6-AS1 overexpression. Further analysis of RNA Pull Down assays for MEK6-AS1 and *MEK6* separately showed that the proteins bound to them were mostly enriched in biological processes related to mRNA splicing, mRNA stability, protein folding and so on. A total of 31 proteins in the two groups were overlapped, among them, NAT10, strongly attracted our attention. NAT10, as an acetyltransferase, has acetyltransferase activity and RNA-binding activity and regulates RNA stability and translation processes [90]. Interestingly, NAT10 was found to act as an RNA cytidine transferase that regulates fatty acid metabolism in tumors [91]. Therefore, we further analyzed the effect of MEK6-AS1 on the stability of *MEK6*, and the results showed that silencing of MEK6-AS1 reduced the stability of *MEK6* mRNA, while overexpression of MEK6-AS1 enhanced it. In addition, interference of *NAT10* reduced both the mRNA stability of MEK6-AS1 and *MEK6*. Consistently, the positive regulation on adipogenic differentiation of hAMSCs by overexpression of MEK6-AS1 was significantly impaired by knockdown of *NAT10*. Our results suggested that as one antisense lncRNA of *MEK6*, MEK6-AS1 potentially enhance the mRNA stability of *MEK6* through NAT10.

Importantly, most lncRNAs have low species conservation and expression abundance, as well as highly specific spatial and temporal expression patterns. This suggests that lncRNAs with clear functions and mechanisms have the potential to not only become human specific biological markers for conditions such as obesity, but also to achieve clinical translation through RNAi therapy [92]. The elaboration of the role and mechanism of MEK6-AS1 on adipogenic differentiation, adipogenesis and fatty acid metabolism provides new ideas and targets for the prevention and treatment of human obesity, MAFLD and other related diseases. Moreover, these findings also enrich the diversity of mechanisms by which lncRNAs regulate stem cell differentiation.

## Conclusions

Here, we identified a human specific lncRNA (lncRNA MEK6-AS1) that has had positional information on UCSC Genome Browser but has not been reported in function. MEK6-AS1 was highly expressed and positively correlated with adipogenic markers in human adipose tissues with high BMI, and was also significantly elevated during hepatic steatosis organoids generation. We have revealed that MEK6-AS1 in human as a novel lncRNA involved in promotes adipogenic differentiation in vitro, enhances adipogenesis in vivo, ameliorates fatty acid metabolism, and inhibits osteogenic differentiation. Mechanistically, MEK6-AS1 works partially through stabilization of *MEK6* mRNA by NAT10. Our findings may provide new comprehension to implications of lncRNA in stem cell biology and offer a novel potential therapeutic target for the prevention and treatment of obesity, MAFLD and other related diseases.

## Supplementary Information

Additional file 1: Figure S1–S10

Additional file 2: Table S1

## Data Availability

All data generated or analyzed during this study are included in this published article and its additional files. The data used in the current study are available from the corresponding authors upon reasonable request.

## Abbreviations

ACACA: Acetyl-CoA carboxylase alpha
ACSL3: Acyl-CoA synthetase long chain family member 3
AFP: Alpha fetoprotein
AGPAT2: 1-Acylglycerol-3-phosphate O-acyltransferase 2
ALP: Alkaline phosphatase
CEBPA: CCAAT enhancer binding protein alpha
COL1A1: Collagen type I alpha 1 chain
COL11A1: Collagen type XI alpha 1 chain
CYP3A4: Cytochrome P450 family 3 subfamily A member 4
DAPI: 4′,6-Diamidino-2′-phenylindole
DGAT2: Acyl CoA: Diacylgycerol acyltransferase 2
ELOVL6: ELOVL fatty acid elongase 6
FABP1: Fatty acid binding protein 1
FABP4: Fatty acid binding protein 4
FADS1: Fatty acid desaturase 1
FFAs: Free fatty acids
FISH: Fluorescence in situ hybridization
FRZB: Frizzled related protein
GAPDH: Glyceraldehyde-3-phosphate dehydrogenase
GPAT3: Glycerol-3-phosphate acyltransferase 3
hAMSCs: Human adipose-derived mesenchymal stem cells
HFD: High-fat diet
HPGD: 15-Hydroxyprostaglandin dehydrogenase
IL-1beta: Interleukin-1beta
IL-6: Interleukin-6
IHC: Immunohistochemistry
iPSCs: Induced pluripotent stem cells
LncRNA: Long non-coding RNA
LPL: Lipoprotein lipase
MAFLD: Metabolic associated fatty liver disease
MCP-1: Monocyte chemoattractant protein-1
MEK6: Mitogen-activated protein kinase kinase 6
MEK6-AS1: Long noncoding RNA MEK6-AS1
MSCs: Mesenchymal stem cells
NAT10: N-acetyltransferase 10
NC: Negative control
OPN: Secreted phosphoprotein 1
PLIN1: Perilipin 1
PLIN2: Perilipin 2
PPARG: Peroxisome proliferator activated receptor gamma
qRT-PCR: Quantitative real-time polymerase chain reaction
RACE: Rapid amplification of cDNA ends
RIP: RNA immunoprecipitation
RUNX2: RUNX family transcription factor 2
SCD: Stearoyl-CoA desaturase
