# Supplementary material for: A novel human specific lncRNA MEK6-AS1 regulates adipogenesis and fatty acid biosynthesis by stabilizing MEK6 mRNA
Source: J Biomed Sci. 2025 Jan 8;32:6. doi: 10.1186/s12929-024-01098-3 (PMC11708274; doi:10.1186/s12929-024-01098-3)
Supplement: Supplementary file 1 — Additional file 1: Figure S1–S10 [file 12929_2024_1098_MOESM1_ESM.doc]

**Additional file 1: Figure S1~S10**

**Figure S1.** Morphological, functional and phenotype identification of hAMSCs. **Figure S2.** Construction and analysis of hepatocyte organoid and hepatic steatosis organoid. **Figure S3.** Full length of lncRNA MEK6-AS1 was verified by RACE assays. **Figure S4.** MEK6-AS1 affected inflammatory factor levels during adipogenic differentiation of hAMSCs. **Figure S5.** Effect of changing expression of MEK6-AS1 on FFAs levels were analyzed in adipogenic-induced cells. **Figure S6.** Effects of MEK6-AS1 on osteogenic differentiation of hAMSCs *in vitro.* **Figure S7.** MEK6-AS1 was effective on adipogenesis of hAMSCs *in vivo* under HFD. **Figure S8.** The impact of MEK6-AS1 on the levels of inflammatory factors during adipogenesis of hAMSCs in vivo. **Figure S9.** *MEK6* also influenced FFAs levels of hAMSCs during adipogenic differentiation. **Figure S10.** MEK6-AS1 was not in direct conjunction with *MEK6*.

**
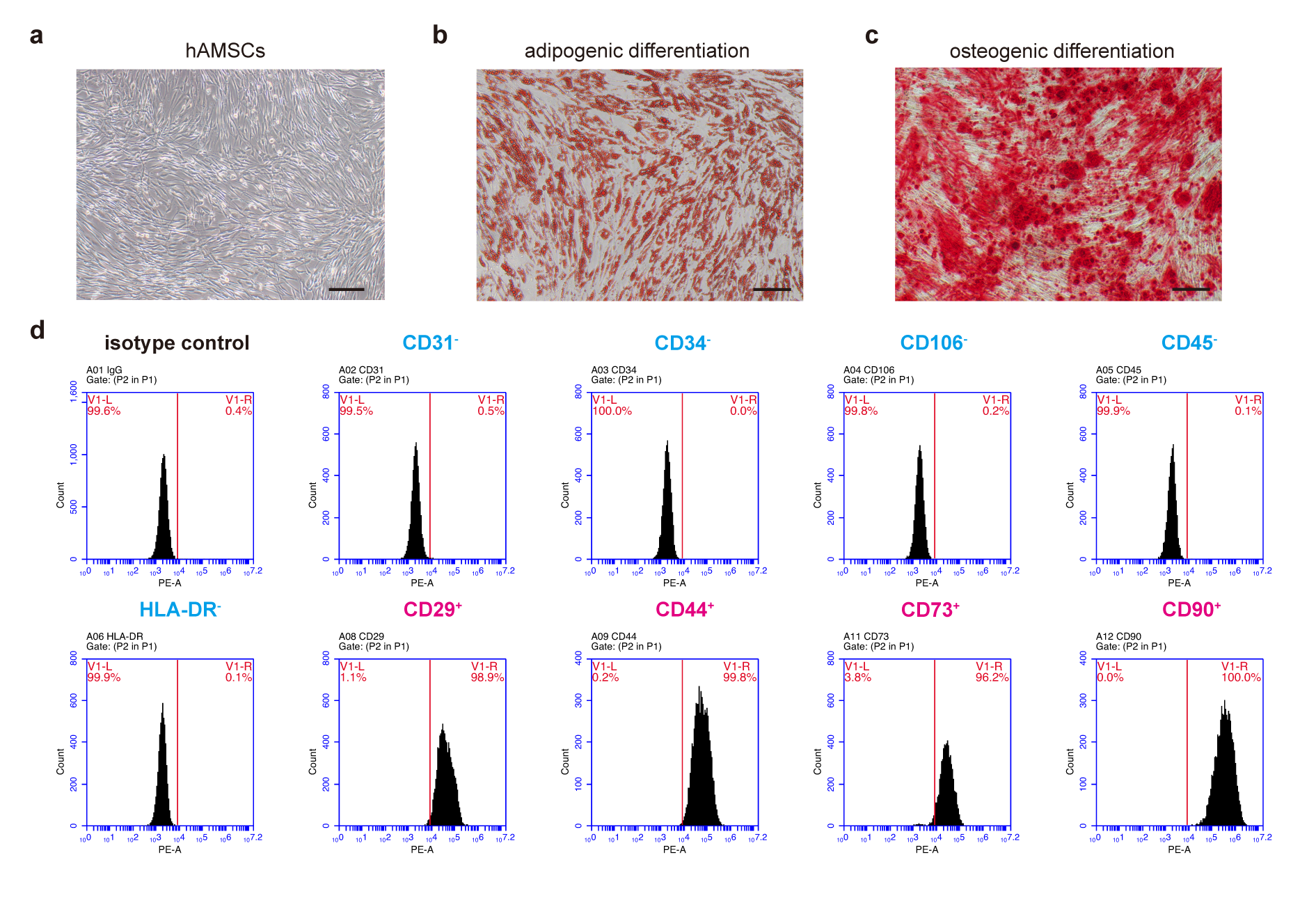
**

**Figure S1. Morphological, functional and phenotype identification of hAMSCs**

(a) Morphology of hAMSCs under the light microscope. Scale bar: 200µm.

(b) Flow cytometry identification of the hAMSC phenotype. hAMSCs were from representative culture at passage 3.

(c) Cells were cultured for adipogenic differentiation for 10 days and visualized with Oil Red O staining (left) or were cultured for osteogenic differentiation for 10 days and assayed with Alizarin Red staining (right). Scale bar: 100µm.


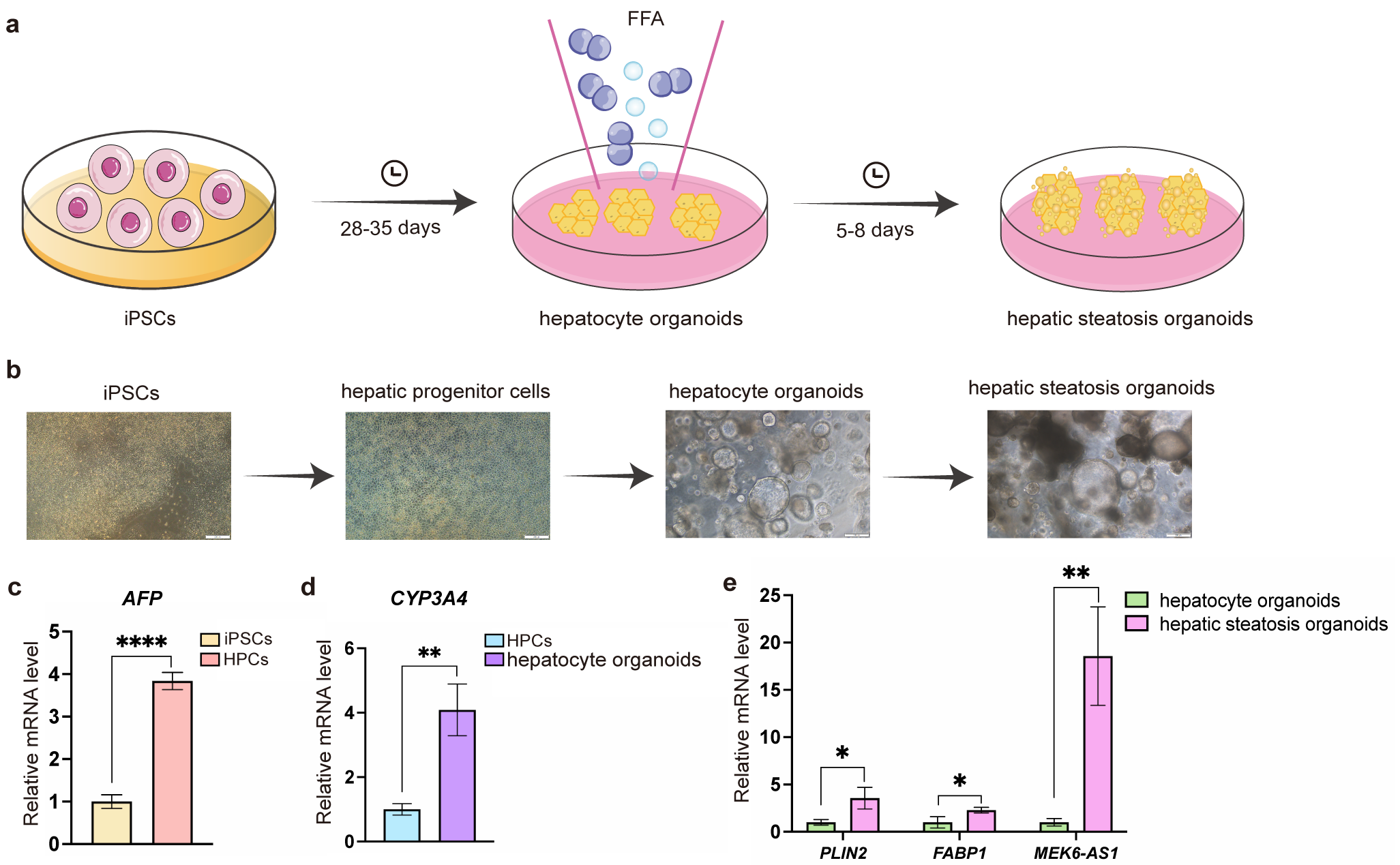


**Figure S2. Construction and analysis of hepatocyte organoids and hepatic steatosis organoids**

(a) Schematic diagram of the induction of iPSCs into organoids.

(b) Morphology of iPSCs, hepatic progenitor cells, hepatocyte organoids and hepatic steatosis organoids under the light microscope. Scale bar: 200µm.

(c) qRT-PCR analysis was used to verify *AFP* expression in different groups of cells.

(d) qRT-PCR analysis was used to verify *CYP3A4* expression in hepatic progenitor cells and hepatocyte organoids.

(e) qRT-PCR analysis was used to verify *PLIN2*, *FABP1* and P6993 expression in different groups of organoids.

*GAPDH* was used as internal control for qRT-PCR. The quantitative data were normalized to *GAPDH*. r: correlation coefficient. When the absolute value of the correlation coefficient approaches 1, it indicates a stronger correlation between the variables. If the correlation coefficient is greater than 0, it indicates a positive correlation between the two. p: p-value.


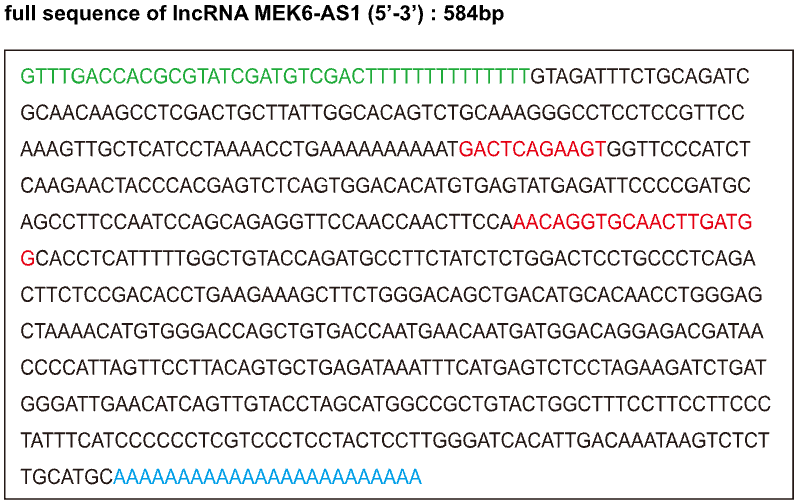


**Figure S3. Full length of lncRNA** **MEK6-AS1 was verified by RACE assays**

The full sequence of MEK6-AS1 shown from 5’ ends to 3’ ends confirmed by RACE. The green bases were the junction sequences of 5' RACE Kit, the red bases were the splice site sequences, respectively.


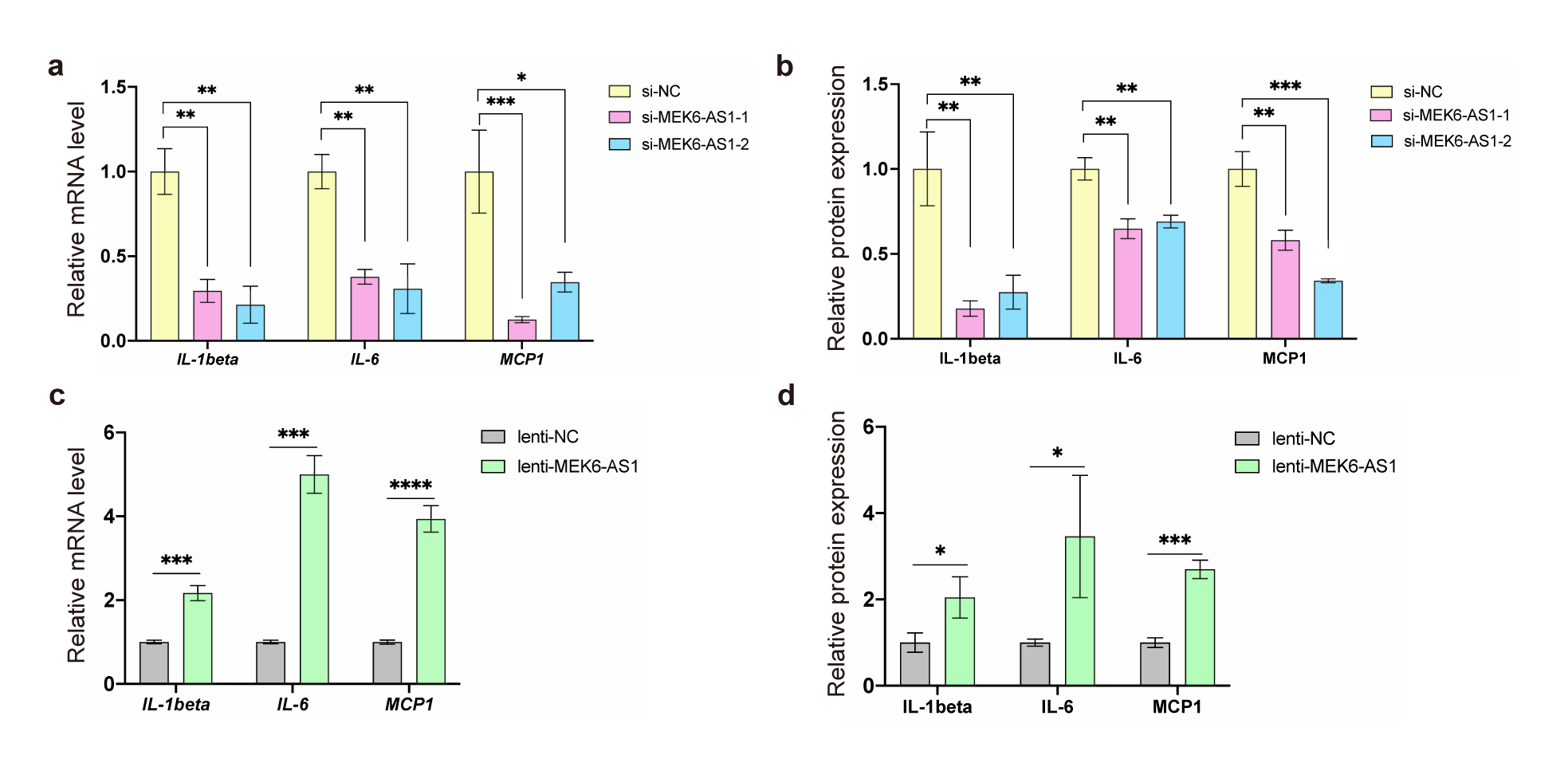


**Figure S4. MEK6-AS1 affected inflammatory factor levels during adipogenic differentiation of hAMSCs**

(a) The expression of key pro-inflammatory cytokine in MEK6-AS1 knockdown and control cells were assessed by qRT-PCR.

(b) Elisa assays were used to tests expression level of IL-1beta, IL-6 and MCP-1.


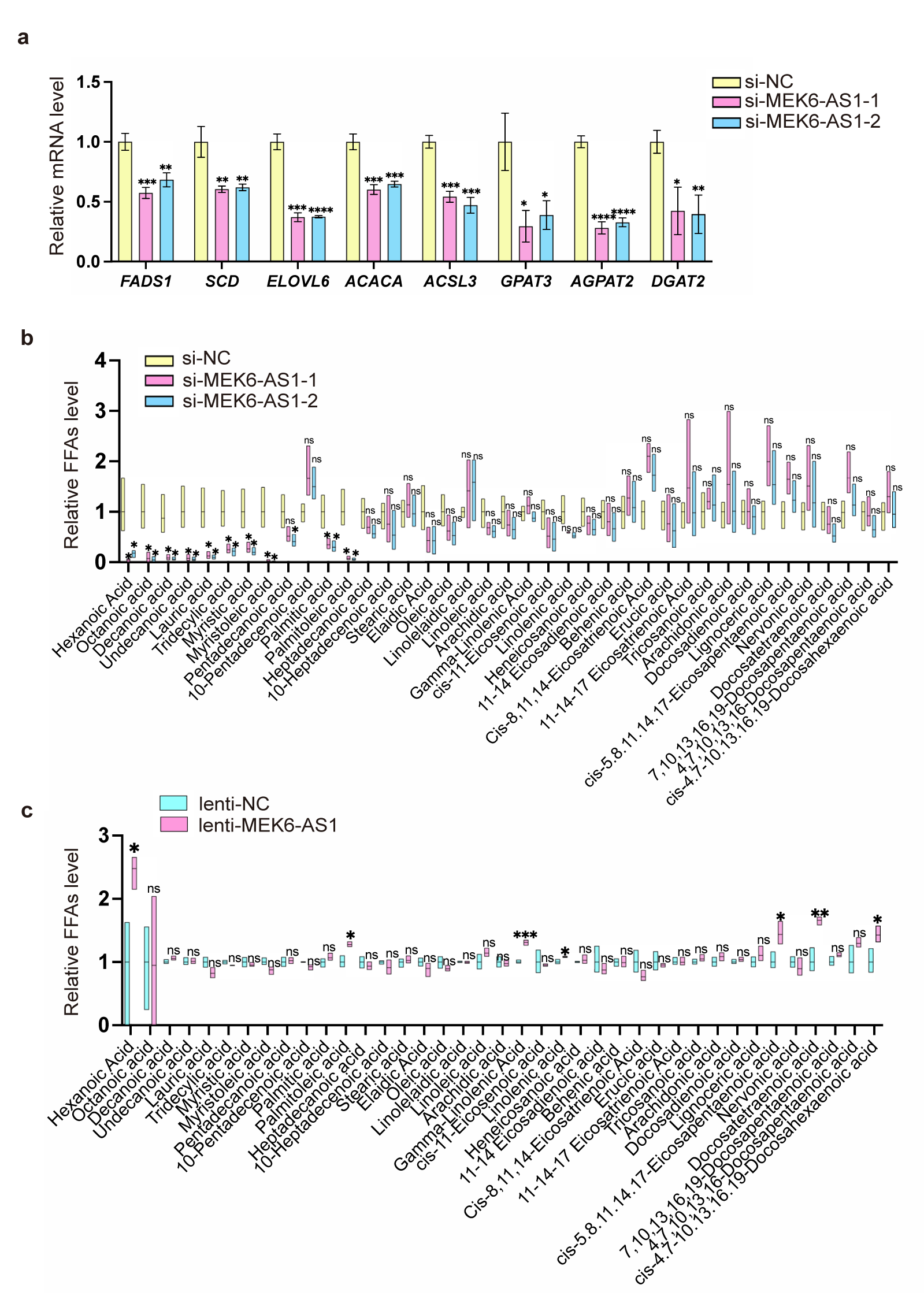


**Figure S5. Effect of changing expression of MEK6-AS1 on FFAs levels were analyzed in adipogenic-induced cells**

(a) The expression of key genes involved in fatty acid metabolism in MEK6-AS1 knockdown and control cells were detected by qRT-PCR.

(b) 39 kinds of FFAs in adipogenic-induced cells after silence of si-MEK6-AS1-1 and si-MEK6-AS1-2.

(c) Mass spectrometry showed that the relative contents of 39 kinds of FFAs in adipogenic-induced cells after overexpression of MEK6-AS1.

*GAPDH* was used as internal control for qRT-PCR and the quantitative data were normalized to *GAPDH*. Data are shown as the mean ± SD. Statistically significant differences were considered as follows: ∗P < 0.05, ∗∗P < 0.01, ∗∗∗P < 0.001, ns: not significant.


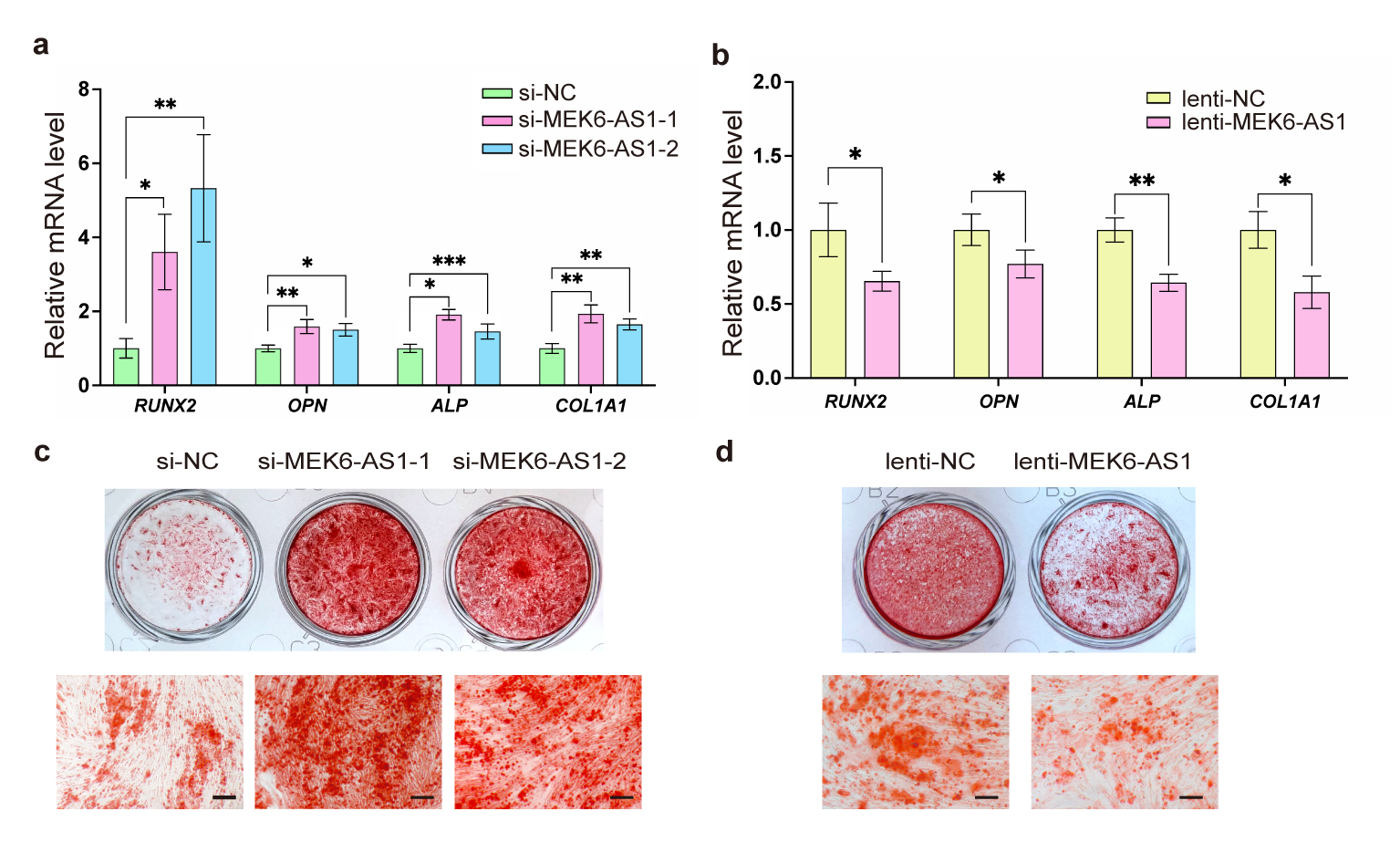


**Figure S6. Effects of MEK6-AS1 on osteogenic differentiation of hAMSCs *in vitro***

(a) The expression of osteogenic markers in hAMSCs with MEK6-AS1 knockdown and in control hAMSCs on day 6 after osteogenic induction were analyzed by qRT-PCR.

(b) Alizarin red staining was used to verify matrix mineralization deposition in cells treated with MEK6-AS1 siRNAs and corresponding control cells on day 10 after osteogenic induction.

(c) The expression of osteogenic markers in cells with MEK6-AS1 overexpression and in control hAMSCs on day 6 after osteogenic induction were analyzed by qRT-PCR.

(d) The gerenation of matrix mineralization deposition in cells on day 10 after osteogenic induction were indicated by Alizarin red staining.

*GAPDH* was used as internal control for qRT-PCR. The quantitative data were normalized to *GAPDH*, n = 3. Data are shown as the mean ± SD. Statistically significant differences were considered as follows: ∗P < 0.05, ∗∗P < 0.01, ∗∗∗P < 0.001. Scale bar: 100µm.


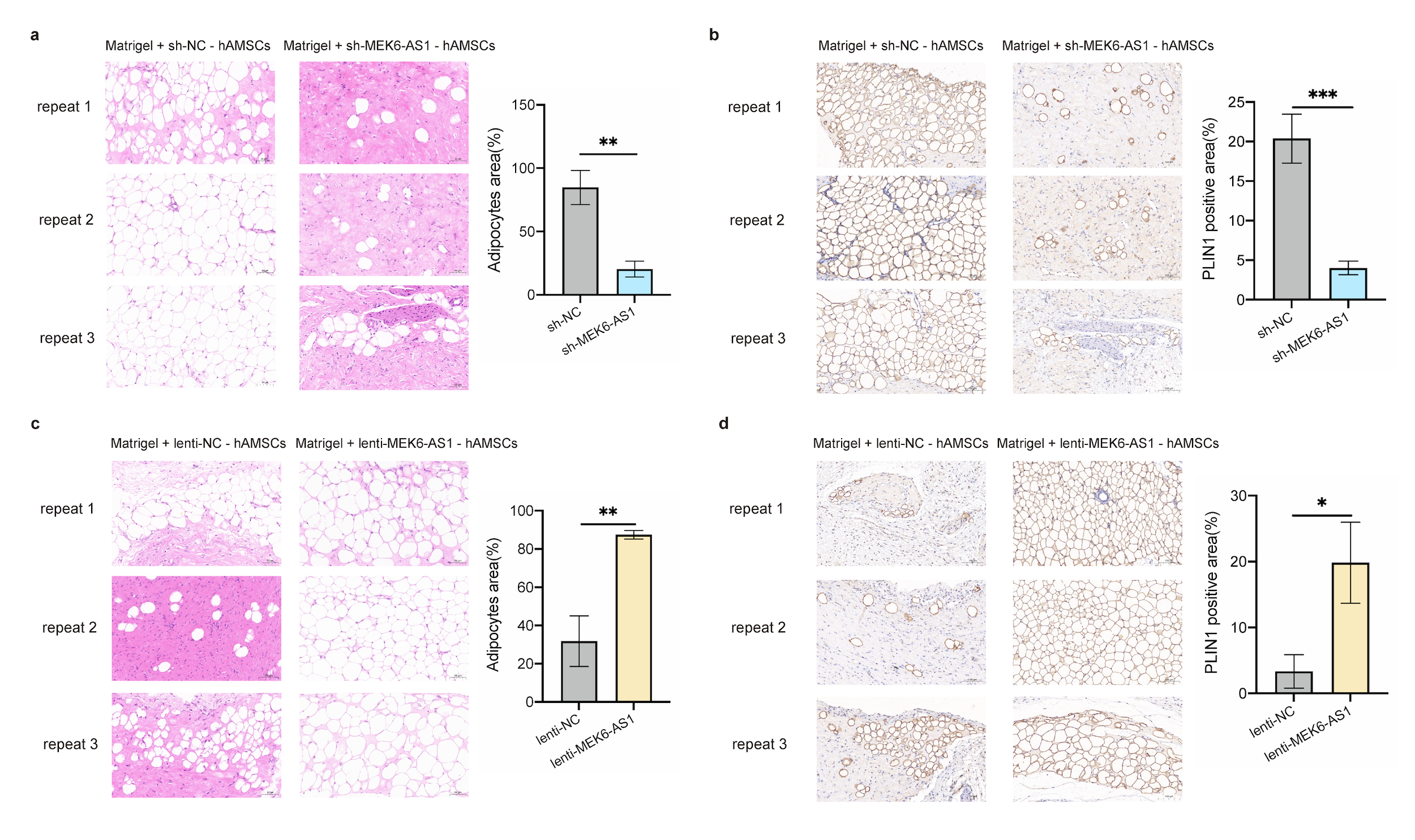


**Figure S7. MEK6-AS1 was effective on adipogenesis of hAMSCs *in vivo* under HFD**

(a and b) Representative H&E staining (a) and PLIN1 IHC staining (b) images for de novo adipogenesis in each group of BALB/c nu/nu mice. n=3. Scale bars: 50µm.

(c and d) Representative H&E staining (c) and PLIN1 IHC staining (d) were performed to validate the de novo adipogenesis in BALB/c nu/nu mice. n=3. Scale bars: 50µm.


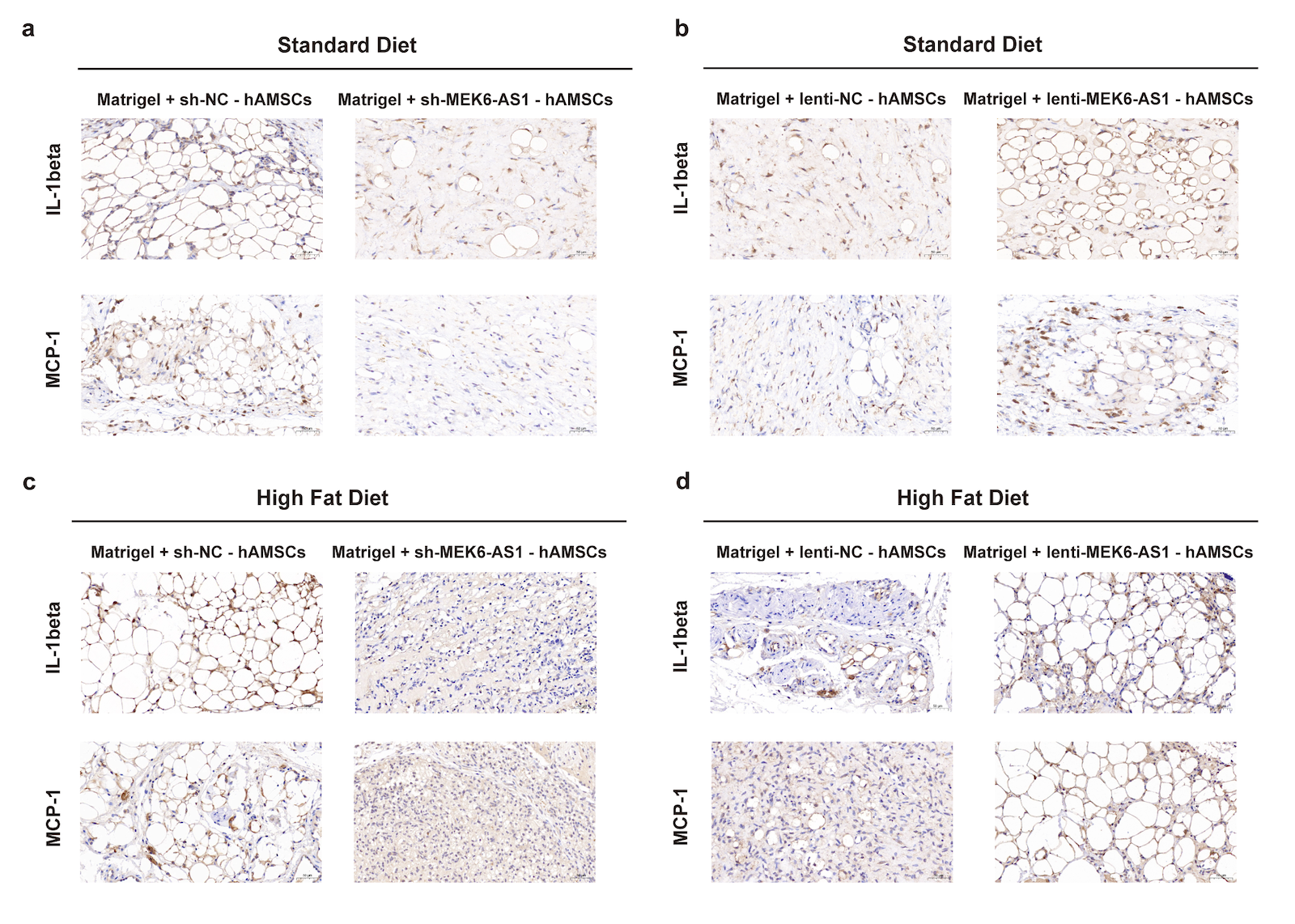


**Figure S8. The impact of MEK6-AS1 on the levels of inflammatory factors during adipogenesis of hAMSCs *in vivo***

(a and b) Representative IHC staining was performed to assess the expression level of IL-1beta (up) and MCP-1 (down) under standard feeding condition. Scale bars: 50µm.

(c and d) Representative IHC staining was used to detect IL-1beta (up) and MCP-1 (down) under HFD condition. Scale bars: 50µm.


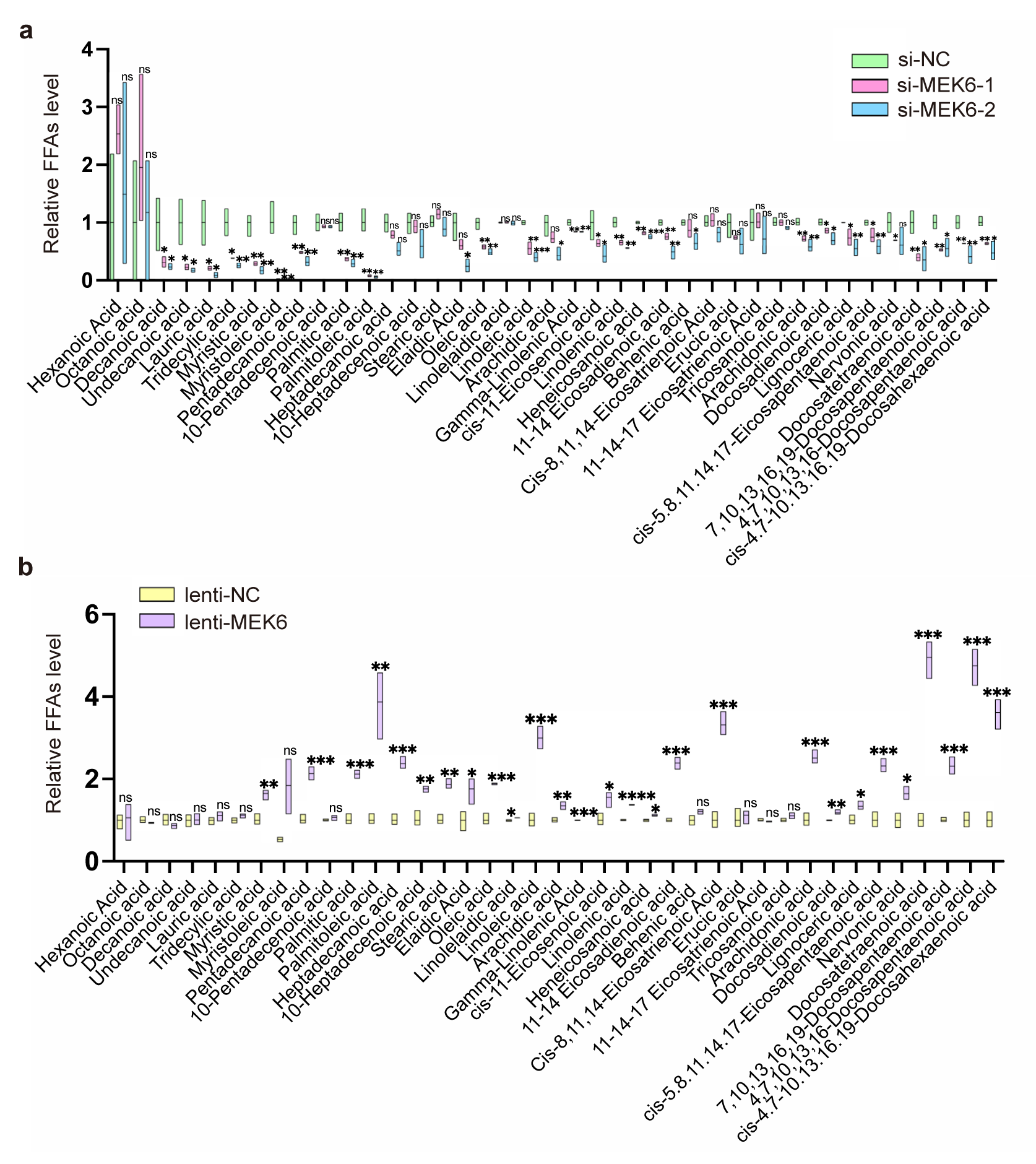


**Figure S9. *MEK6* also affected FFAs levels of hAMSCs during adipogenic differentiation**

(a) 39 kinds of FFAs in adipogenic-induced cells after silence of si-MEK6-1 and si-MEK6-2.

(b) Mass spectrometry showed that the relative contents of 39 kinds of FFAs in adipogenic-induced cells after overexpression of *MEK6*.

Data are shown as the mean ± SD, n=3. Statistically significant differences were considered as follows: ∗P < 0.05, ∗∗P < 0.01, ∗∗∗P < 0.001, ns: not significant.


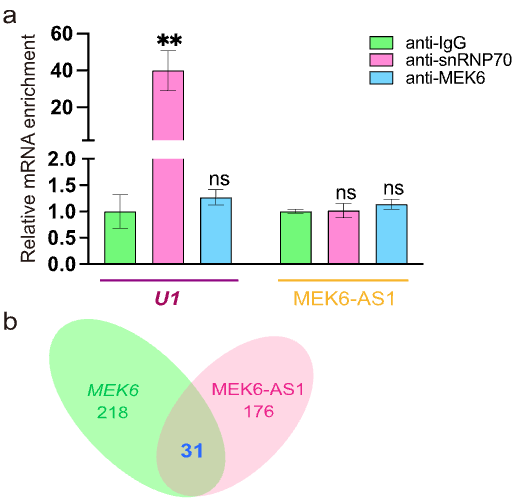


**Figure S10. MEK6-AS1 was not in direct conjunction with *MEK6***

(a) MEK6-AS1 might bind 207 proteins (pink) and *MEK6* mRNA might bind 249 proteins (green). A total of 31 proteins in the two groups were overlapped (blue).

(b) RNA Binding Protein Immunoprecipitation (RIP) experiments revealed that MEK6-AS1 might not bind directly to *MEK6*. *U1* which bound with snRNP70 was used for positive control by guidance of the RIP Kit.

*GAPDH* was used as internal controls for qRT-PCR. The quantitative data were normalized to *GAPDH*, n = 3. Data are shown as the mean ± SD. Statistically significant differences were considered as follows: ∗∗P < 0.01, ns: not significant.
