# Supplementary material for: A novel human specific lncRNA MEK6-AS1 regulates adipogenesis and fatty acid biosynthesis by stabilizing MEK6 mRNA
Source: J Biomed Sci. 2025 Jan 8;32:6. doi: 10.1186/s12929-024-01098-3 (PMC11708274; doi:10.1186/s12929-024-01098-3)
Supplement: Supplementary file 2 — Additional file 2: Table S1 [file 12929_2024_1098_MOESM2_ESM.doc]

**Additional file 2: Table S1. The sequences of primers used in this study.**

| Target | Primer sequence (5’-3’) |
| --- | --- |
| MEK6-AS1 | F: GTGAGTATGAGATTCCCCGATG |
|  | R: AGGAGTCCAGAGATAGAAGGC |
| *MEK6* | F: GAAGCATTTGAACAACCTCAGAC |
|  | R: CCTGGCTATTTACTGTGGCTC |
| *AFP* | F: CTGCAATTGAGAAACCCACTG |
|  | R: TTCCCTCTTCACTTTGGCTG |
| *CYP3A4* | F: ACCAGTGGAAAACTCAAGGAG |
|  | R: TGATCACATCCATGCTGTAGG |
| *PPARG* | F: TGAACGTGAAGCCCATCGAG |
|  | R: CTTGGCGAACAGCTGAGAGG |
| *FABP1* | F: CTGGGTCCAAAGTGATCCAAA |
|  | R: TCCAACTGAACCACTGTCTTG |
| *FABP4* | F: ACTGGGCCAGGAATTTGACG |
|  | R: CTCGTGGAAGTGACGCCTT |
| *PLIN1* | F: CCATGTCCCTATCAGATGCCC |
|  | R: CTGGTGGGTTGTCGATGTC |
| *PLIN2* | F: GAGTGGAAAAGGAGCATTGGA |
|  | R: CCTTGGATGTTGGACAGGAG |
| *CEBPA* | F: GCGCAAGAGCCGAGATAAAG |
|  | R: CGGTCATTGTCACTGGTCAACT |
| *GAPDH* | F: GGTCACCAGGGCTGCTTTTA |
|  | R: GGATCTCGCTCCTGGAAGATG |
| *LPL* | F: TCATTCCCGGAGTAGCAGAGT |
|  | R: GGCCACAAGTTTTGGCACC |
| *NAT10* | F: GCCTCTTGTAAGAAGTGTCTCG |
|  | R: TCTTTTCAGAGATGCCCTCGAT |
| 5' RACE R1 | GCTGCATCGGGGAATCTCAT |
| 5' RACE R2 | CACATGTGTCCACTGAGAC |
| 5' RACE RT | CATCAAGTTGCACCTGTTTG |
| 3' RACE F1 | GTCTCAGTGGACACATGTG |
| 3' RACE F2 | TATGAGATTCCCCGATGCAGC |
| si-NAT10-1 | F: GCAUUUGGGUACUCCAAUATT |
|  | R: UAUUGGAGUACCCAAAUGCTT |
| si-NAT10-2 | F: GGGCCAGGCUGAACUAGUUTT |
|  | R: AACUAGUUCAGCCUGGCCCTT |
| si-MEK6-1 | F: CCAAGGCUUGCAUUUCUAUTT |
|  | R: AUAGAAAUGCAAGCCUUGGTT |
| si-MEK6-2 | F: GCUCAUGGAUACAUCACUATT |
|  | R: UAGUGAUGUAUCCAUGAGCTT |
| si-MEK6-AS1-1 | F: GCUGUGACCAAUGAACAAUTT |
|  | R: AUUGUUCAUUGGUCACAGCTT |
| si-MEK6-AS1-2 | F: GUGGUUCCCAUCUCAAGAATT |
|  | R: UUCUUGAGAUGGGAACCACTT |
